# Synaptic dysfunction in neurodegenerative and neurodevelopmental diseases: an overview of induced pluripotent stem-cell-based disease models

**DOI:** 10.1098/rsob.180138

**Published:** 2018-09-05

**Authors:** Era Taoufik, Georgia Kouroupi, Ourania Zygogianni, Rebecca Matsas

**Affiliations:** Laboratory of Cellular and Molecular Neurobiology–Stem Cells, Department of Neurobiology, Hellenic Pasteur Institute, 127 Vassilissis Sofias Avenue, 11521 Athens, Greece

**Keywords:** neurodevelopmental diseases, Parkinson's disease, Huntington's disease, synaptopathy, organoids

## Abstract

Synaptic dysfunction in CNS disorders is the outcome of perturbations in physiological synapse structure and function, and can be either the cause or the consequence in specific pathologies. Accumulating data in the field of neuropsychiatric disorders, including autism spectrum disorders, schizophrenia and bipolar disorder, point to a neurodevelopmental origin of these pathologies. Due to a relatively early onset of behavioural and cognitive symptoms, it is generally acknowledged that mental illness initiates at the synapse level. On the other hand, synaptic dysfunction has been considered as an endpoint incident in neurodegenerative diseases, such as Alzheimer's, Parkinson's and Huntington's, mainly due to the considerably later onset of clinical symptoms and progressive appearance of cognitive deficits. This dichotomy has recently been challenged, particularly since the discovery of cell reprogramming technologies and the generation of induced pluripotent stem cells from patient somatic cells. The creation of ‘disease-in-a-dish’ models for multiple CNS pathologies has revealed unexpected commonalities in the molecular and cellular mechanisms operating in both developmental and degenerative conditions, most of which meet at the synapse level. In this review we discuss synaptic dysfunction in prototype neurodevelopmental and neurodegenerative diseases, emphasizing overlapping features of synaptopathy that have been suggested by studies using induced pluripotent stem-cell-based systems. These valuable disease models have highlighted a potential neurodevelopmental component in classical neurodegenerative diseases that is worth pursuing and investigating further. Moving from demonstration of correlation to understanding mechanistic causality forms the basis for developing novel therapeutics.

## 1. Introduction

Central nervous system (CNS) disorders are a group of diseases with significant socioeconomic impact and growing relevance due to the increase in life expectancy of the world population. Since only symptomatic or palliative therapies are currently available for most of these diseases, the development of innovative therapeutic strategies is an unmet need. CNS disorders, traditionally dichotomized between early-onset neurodevelopmental and late-onset neurodegenerative diseases, are associated with dysfunction of neuronal activity due to perturbations at the synapse level [1]. They may therefore be collectively regarded as diseases of the synapse or synaptopathies. Synaptic defects are causally associated with early appearing neurological diseases, including autism spectrum disorders (ASD), schizophrenia (SCZ) and bipolar disorder (BP). On the other hand, in late-onset degenerative pathologies, such as Alzheimer's (AD), Parkinson's (PD) and Huntington's (HD) diseases, synaptopathy is thought to be the inevitable end-result of an ongoing pathophysiological cascade. However, understanding the initiation and contribution of synaptic dysfunction in neurological disorders has been challenging because of (i) limited and usually late-stage access to human tissue, and (ii) inadequate recapitulation of key features of the human diseases in existing experimental animal models. Recent advances in cell reprogramming technologies that allow generation of human induced pluripotent stem cells (hiPSCs) [2] from somatic cells of patients with a variety of diseases have opened new perspectives for studying the pathogenesis of CNS disorders. The establishment of robust protocols for directing the differentiation of hiPSCs into various neuronal cell types has permitted disease-in-a-dish modelling and analysis of the phenotypic characteristics of numerous CNS pathologies. More recently, the development of three-dimensional (3D) organoid cultures has created new possibilities for studying disease emergence and progression in the closest situation to the human brain [3]. Due to these revolutionizing technologies it is now possible to shed light into cellular and molecular mechanisms underlying neuronal dysfunction in patient cells and follow over time the emergence of disease phenotypes, particularly those appearing early.

In this review we discuss findings from hiPSC-based cellular models of neurodevelopmental neuropsychiatric disorders, including ASD and SCZ, and of neurodegenerative diseases, focusing on HD and PD. We present evidence that support synaptopathy as a central feature of these pathologies and raise the intriguing hypothesis that defects in synaptic function may comprise an early and, possibly, triggering event in the pathogenesis of not only neurodevelopmental but also neurodegenerative diseases. The experimental challenges and limitations of using hiPSC-based models for understanding synaptic dysfunction in neurological diseases are also considered, together with the potential of overcoming these significant drawbacks to gain a deeper understanding of disease mechanisms and develop effective therapeutic strategies.

## 2. Structural and molecular overview of ‘one healthy synapse’

The synapse comprises the major information transfer unit in the nervous system, and proper brain function relies on the accurate establishment of synaptic contacts during development. As a number of mutations in synaptic proteins have been linked to neurodevelopmental disorders [4] and impaired function at various sites of the synapse comprises a dominant feature of neurodegenerative diseases [5], it is important to present here a short overview of synaptogenesis and synapse organization in the healthy nervous system, and identify key molecules that orchestrate these processes.

**Figure 1.**
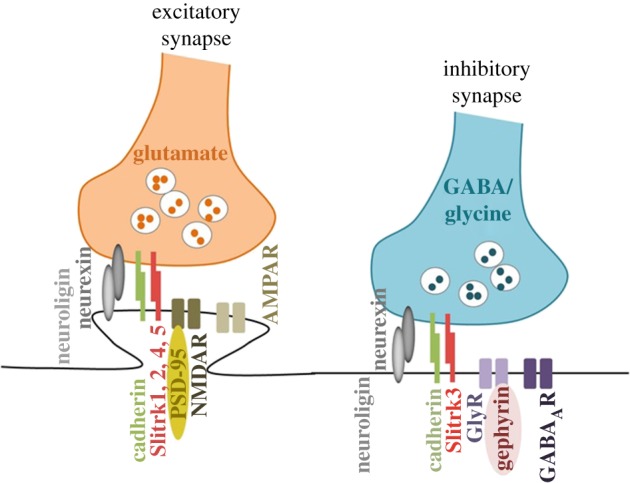
Schematic diagram depicting the molecular organization of excitatory and inhibitory synapses. The excitatory neurotransmitter glutamate is released from presynaptic neurons and binds to glutamate receptors NMDA and AMPA located in postsynaptic dendritic spines. Inhibitory neurotransmitters (gamma aminobutyric acid GABA or glycine) are released from presynaptic neurons and bind to GABA_A_ and glycine receptors clustered by gephyrin, the best-known inhibitory PSD protein. Synapse-organizing adhesion molecules include neurexins and neuroligins, cadherins, SLITRKs and others.

CNS synapses are intercellular junctions between neurons that transmit action-potential encoded information [6]. They are largely divided into *electrical*, which allow direct transfer of charged ions and small molecules through pores known as gap junctions, and *chemical*, which transfer electrical activity uni-directionally from one neuron to another via chemical mediators, the neurotransmitters [7]. Depending on the nature of these molecules, synapses are further subdivided in excitatory and inhibitory, with glutamatergic and GABAergic synapses being the dominant sources of excitation and inhibition, respectively, throughout the mammalian brain (figure 1). Excitatory synapses are mainly located at the tip of tiny dendritic protrusions, the dendritic spines, while inhibitory synapses are formed on the shaft of dendrites or on cell bodies and axon initial segments [8]. Despite distinct morphology, function and molecular composition, the overall organization of the synapse comprises a presynaptic terminal loaded with neurotransmitter-containing vesicles, perfectly juxtaposed to the postsynaptic compartment, which is decorated with an array of surface receptors responsive to neurotransmitter release. The two compartments are held together by synaptic cell adhesion molecules [9–11].

The presynaptic terminal is formed early during development when the navigating axon projects to distant target regions and, in the presence of appropriate signals, the undifferentiated portion of the axoplasm undergoes changes to become a specialized area of clustered synaptic vesicles (SVs) [6,9]. Despite minor differences between organisms and synapse type, all presynaptic terminals share an identical structure. Microscopically, the membrane region where SVs are clustered to be exocytosed is spotted as an electron-dense thickening designated as the presynaptic active zone. This area forms an intrinsic part of the synaptic vesicle release machinery where docking and priming of synaptic vesicles occurs, followed by recruitment of Ca^2+^ channels to allow fast synchronous excitation/release coupling. The molecular composition of the active zone has been analysed extensively [12] and the number of proteins associated with the pre-synaptic terminal is significantly higher than anticipated, with around 450 proteins being identified so far [6,13–15]. Apart from the highly conserved protein complex of Rab3-interacting molecules (RIMs), RIM-binding proteins, α-liprin, Munc13 and ELKS, that is enriched in the active zone, other pre-synaptic molecules include piccolo and bassoon, synapsin, synaptophysin, synaptogyrin and SV2, components of the SNARE-complex such as VAMP2/synaptobrevin, small GTPases, neurotransmitter transporters such as vGLUT1 and VGAT, channel proteins such as proton pumps, calcium sensors and axonal trafficking proteins such as kinesins and dyneins. Synapse formation is initiated in different ways; when at close proximity, axons and dendrites will interact by extension of dendritic filopodia or the axonal branches, including the growth cone [16]. In the conventional synaptogenesis model, the presynaptic material is recruited and clustered on sites of axodendritic contact in an inherited way, but complete differentiation of a stable presynaptic terminal requires contact with a post-synaptic partner and is dependent on synaptogenic cues that are either trans-synaptic or soluble synaptic molecules.

The trans-synaptic adhesion molecules organize the synaptic junctions bridging the synaptic cleft. Rather than just a gap, this area is a protein-rich environment initially identified as an electron-dense material in the extracellular space [17]. We now know that this area has extensive bridging fibrils anchored to intra-membrane particles as well as fibril-like structures oriented parallel to the synaptic membranes [18,19]. Insights into the molecular identity of the synaptic cleft complexes has identified that their role is not only to physically connect the pre- and post-synaptic compartment but also to mediate recognition and signalling processes that are essential for the establishment, specification and plasticity of synapses. Such synapse-organizing adhesion molecules include neurexins and neuroligins, cadherins, integrins, Ig-domain proteins SynCAMs, receptor phosphotyrosine kinases and phosphatases such as ephrins and Rho GTPases, and leucine-rich repeat (LRRTMs) proteins such as SLITs, SALMs and netrins [11,20,21]. Specific adhesion proteins seem to induce functional pre-synaptic release sites while others activate post-synaptic specializations, however this process largely depends on the synapse type and has not been well characterized [22].

Opposed to the pre-synaptic zone is the post-synaptic side of the synapse (PSD), a disc-like structure specialized to receive the neurotransmitter signal released from the presynaptic terminal and transduce it into electrical and biochemical changes in the post-synaptic cell [23]. The cardinal functional components of post-synaptic specialization into excitatory or inhibitory synapses are the ionotropic receptors (ligand-gated channels) for glutamate and γ-aminobutyric acid (GABA), respectively. These receptor channels are concentrated at the post-synaptic membrane and are embedded in a dense and rich protein network composed of anchoring and scaffolding molecules, signalling enzymes, cytoskeletal components, as well as other membrane proteins. Overall more than 400 protein components have been described to date [10].

The excitatory and inhibitory synapses differ significantly in morphology, composition and organization. Due to its large abundance and distinctive structure, the glutamatergic synapse mediated by NMDA and AMPA glutamate receptors has been studied most extensively [23]. As with the synaptic cleft, the post-synaptic side has a striking architecture patterned in three dimensions where glutamate receptor subtypes have a distinctive distribution, with AMPARs enriched in the extrasynaptic membrane and NMDARs found towards the centre [24]. This compartmentalization is facilitated by the membrane-associated guanylate kinase scaffold proteins (MAGUK), typified by PSD-95 in most mature synapses. Other MAGUK proteins include Shank3, Homer1α and GKAP2, which together with the glutamate receptors form ‘supercomplexes’ that act as seeds for further structures enriched in the highly abundant CamKIIa and CamKIIb kinases [22]. The GABAergic post-synaptic side is less dense than the glutamatergic, suggesting a much less elaborate specialization [25]. The major receptors identified are GABA_A_ and glycine receptors clustered by gephyrin, the best-known inhibitory PSD protein [26]. Analysis of the inhibitory and excitatory PSD shows a molecular overlap; yet, unique components remain to be identified for understanding circuit wiring and function, as well as cognitive disorders.

The establishment of stable functional synapse includes three major stages: the initial synapse establishment upon contact of an axon with a target cell, organization of synapse components to construct the canonical synaptic machinery and, last, the specification of synapse properties to confer the unique characteristics for any given synapse. This is a multi-step process orchestrated by numerous molecules acting in a highly controlled manner in space and time [27], occurs both during development and in the adult brain, and is a dynamic, activity-dependent process that regulates the balance between excitatory and inhibitory signals in neural circuits [28].

## 3. Synaptopathy: a common denominator of neurological diseases

Synapses operate as ensembles within defined neural networks to direct the level of neuronal activity, critical for learning, memory and behaviour, therefore it is not surprising that synaptic disturbances can have detrimental consequences. Synaptic dysfunction results from alterations in cell-intrinsic molecular mechanisms or from changes in biochemical processes occurring in the surrounding environment [29].

An early or late synaptic dysfunction is a common denominator of a number of diseases, collectively termed synaptopathies [5,29]. This increasingly popular term attempts to bring under the same umbrella quite diverse pathologies. These include neurodegenerative diseases, such as PD, HD, AD and prion pathologies, characterized by progressive loss of neural tissue that is accompanied by a slow decline in cognitive and behavioural function. This has been largely attributed to the slow accumulation of protein aggregates in neurons that might differ in composition depending on the pathology, but have similar detrimental consequences to neuronal integrity [30]. Pathological species of specific ‘hallmark-of-disease’ proteins such as alpha-synuclein (αSyn) in PD [31], Tau in AD [32] and Huntingtin (HTT) in HD [33] accumulate and mislocalize in diseased neurons. This results to proteostasis imbalance that affect greatly synaptic terminal composition, organization and function by various mechanisms including local proteins synthesis and clearance, described elsewhere in detail [34–37]. The correlation between pathological protein aggregation and neuronal dysfunction serves as the basis for the development of anti-aggregation compounds that have shown promising results in pre-clinical studies [37–39].

Neurodevelopmental disorders, including ASD, intellectual disability (ID) and SCZ, characterized by abnormal behavioural or cognitive phenotypes originating either *in utero* or during early post-natal life, have also been associated with synaptic defects mainly due to the preponderance of penetrant mutations associated with synaptic structure and function [40] and dendritic spine alterations in post-mortem tissue [41].

Evidence for synaptic dysfunction in neurological diseases has been largely relying on three traditional approaches: genetic studies in patients, analysis of post- mortem diseased tissue and animal models. The genetic studies have confirmed high heritability and risk within-family for a number of neurodevelopmental and degenerative disorders. Genome sequencing has identified a large number of disease-associated risk loci, and complementary transcriptomic analysis has aided assessment of functional consequences of some of these genetic variants; however, they cannot provide answers relating to primary or secondary disease phenotypes. In a similar manner, the cellular and molecular analysis of disease-relevant post-mortem tissue reveals important clues for disease progression and endpoint characteristics, but not for early or initiating events, which might include alterations in circuit formation and function during pre-natal stages of development. The next best tool available, animal models, have failed to show significant predictive validity for drug discovery. This could be due to their inability to simulate unique human functions, and therefore recapitulate key manifestations characterizing a particular disorder. Especially in neurological diseases, modelling cognitive dysfunction and psychiatric behaviour has been challenging, with limited success [42]. Despite the contribution of these approaches in understanding that synaptopathy lies at the core of many neurological diseases, the distinction between primary and secondary synaptic phenotypes and how these eventually lead to specific neurological symptoms remain unknown. At the same time the dysregulation of common cellular pathways between neuropsychiatric conditions and late-onset neurodegenerative disorders has been overlooked due to the very different nature of these pathologies and time of clinical onset.

However, as we gain a deeper insight into fundamental mechanisms of neurogenesis, synapse formation, maintenance and plasticity, and develop novel systems and tools for studying early pathogenic events for late-appearing neurological diseases, the classical lines of dichotomy become blurred and an emergent picture suggests more complex and probably overlapping mechanisms of synaptic dysfunction.

## 4. Investigating synaptic dysfunction in hiPSC-based models of neurological disorders

Even though clinical symptoms of neurological diseases can appear in childhood, early adulthood or late adulthood, the time of initiation of the pathological cascades remains a black box and there is evidence to support neuronal circuitry perturbations during early neuronal development despite later manifestation of clinical symptoms. To investigate these critical pathological events in the developing human brain or in early childhood seemed unimaginable until the recent era of cell reprogramming technologies and advances in organogenesis.

### 4.1. Human induced pluripotent stem cells: reprogramming and differentiation

Human induced pluripotent stem cells have similar self-renewal and pluripotency properties as human embryonic stem cells but are derived from adult somatic cells, such as skin fibroblasts, keratinocytes, dental pulp or blood [43], and are therefore devoid of accessibility and ethical issues. Reprogramming of somatic cells is achieved by forced expression of key pluripotency genes such as OCT4, SOX2, c-MYC and KLF4 in somatic cells, where they initiate a self-regulatory loop that converts adult cells to an embryonic-like state and maintains pluripotency [2]. The mode of gene delivery varies from viral transduction to viral-free systems and the concurrent introduction of small molecules that increase reprogramming efficiency [44]. The process is highly specific, involves activation of developmental programmes, is largely inefficient and is affected by many factors, including cell cycle regulators and bioenergetics [43].

Differentiation of hiPSC into neuronal cells is achieved with neural induction mediated by specific morphogenetic factors that are normally expressed in the developing human brain [45,46]. The remarkable feature of this differentiation process *in vitro* is that it mimics, to a large extent, human brain development. hiPSCs are directed to acquire a neuroectodermal fate where induction of a regional neuroepithelial phenotype is achieved by specific patterning factors that in turn prompt the expression of master regulatory transcription factors, characteristic for the desired type of neural progenitor cells (NPCs). This progenitor pool can be expanded and stored providing a valuable source for experimentation. Disease-relevant hiPSC-based systems are obtained through guided differentiation of these NPCs usually towards neural cell types associated with the disease of interest. This process is known as ‘monolayer differentiation’, has been successful in generating various neuronal subtypes including cortical excitatory projection neurons [45] and inhibitory interneurons [47], hippocampal granule neurons [48], dopaminergic neurons [49] and striatal medium spiny neurons [50], and has proved very useful in a multitude of studies modelling neurological diseases that will be discussed in detail below.

### 4.2. Functional characterization of hiPSC-derived neurons

Variations in 2D culture protocols have been developed and successfully led to rapid, more than 95% efficiency of neuronal differentiation with concurrent generation of highly enriched neuronal subpopulations. However, when modelling synaptopathies, the challenge is to produce neurons with functional properties [51]. Currently there is no single ‘gold standard’ to determine synaptic dysfunction in hiPSC-derived neurons and conclusive information can only be achieved by combining morphological, molecular and electrophysiological tools to analyse the expression of synapse-associated molecules, formation of synaptic contacts, electrophysiological properties such as voltage-gated inward and outward currents, firing of action potentials (AP) in response to current injection, responses to glutamate and GABA and spontaneous synaptic activity [51,52]. The majority of hiPSC-based studies describe presence of functionally mature neurons at various stages during the differentiation process, however most often they present recordings of individual neurons without addressing neuronal connectivity issues. As synaptopathy is arising from neuronal communication defects, being able to assess the overall functionality of the 2D culture circuitries is essential. Novel region-specific differentiation protocols [53], forced expression of transcription factors such as Neurogenin2 [54], generation of novel media formulations [55], automated reprogramming [56], and inclusion of human glial cell types [57,58] that support synaptic development and pruning [44] are facilitating the formation of higher-order neural networks *in vitro* that reflect stages and activity patterns of respective developing regions *in vivo*.

A good example of such an approach is presented by Kirwan *et al.* [59], who performed an extensive characterization of functional development of cortical neuronal assemblies *in vitro* enriched in astrocytes. Using calcium imaging to allow the detection of neuronal activity across the whole culture and at single-cell resolution, cortical neurons matured slowly over time with the same stereotypical order that occurs in cerebral cortex *in vivo*, as shown by the level of synchronicity, inter-burst intervals and frequency. This was accompanied by a gradual morphological maturation of excitatory dendritic spines and expression of neurotransmitter receptors suggestive of mature synapses. A most important finding is that the use of single-neuron trans-synaptic tracing of pseudotyped rabies virus demonstrated large number of neurons with few connections and a small number of highly connected cells following the same connectivity pattern of developing cortical neurons. Manipulating specific cell types within circuits is also highly informative on synaptic activity of hiPSC-derived neurons. Optogenetic stimulation of host rodent cells revealed an immature phenotype of grafted hiPSC-derived mDA neurons suggestive of limited functional synaptic connections [60,61]. The use of sensors to image neural activity *in vitro* is not currently widely applicable as most 2D systems rely on enriched single neuronal subtypes, usually representing the type affected in the specific neurological disorder. However as microfluidic systems develop to allow reconstitution of neuronal networks on chips [62], fluorescent sensors will be valuable to manipulate synaptic activity in hiPSC-derived neuronal networks.

Electrophysiological maturity of neurons is strongly correlated with the expression of transcripts associated with AP firing, channels, synaptic receptors, synaptic stabilizers and a number of synaptic activity-dependent genes. RNA-Seq analyses of whole networks or of individual neurons are providing signatures associated with neuronal type and maturation state. Excitatory input to hiPSC-derived neurons induced human immediate early genes in a lineage-specific fashion, as the synaptic activity-induced gene programmes were highly enriched for genes that are involved in attention deficit/hyperactivity disorder, episodic memory formation and long-term memory [63]. This suggests that the hiPSC-derived neurons activate gene developmental processes associated with higher cognitive functions in a highly conserved manner, and genetic differences that might lead to various neurological diseases and have an impact on synaptic activity-dependent transcription could result in relevant phenotypes *in vitro.* In support of this approach, a recent study showed that activity-dependent changes in gene expression of SZC neurons after depolarization with potassium chloride [64] were largely attenuated.

Collectively these approaches clearly show that hiPSC-derived neurons are capable of forming functional synapses and participate in circuits despite the fact that they do not reach adult neuron characteristics in culture. The fetal nature of hiPSC-derived neural cells, though, must be taken into consideration especially when attempting to simulate late-onset diseases [65]. Microarray gene expression profiles of hiPSC forebrain NPCs and 6-week neuronal cultures shared the most similarity with first-trimester fetal brain tissue [66]. This study showed a remarkable resemblance in cortical and subcortical forebrain transcriptome identity but also a persistent fetal-like phenotype regardless of the maturation stage of the culture. Alternative approaches to generate neurons by direct conversion of somatic cells to accelerate maturation by retaining genetic hallmarks of ageing [44] or by inducing cellular ageing by the ectopic expression of gene products [67] or addition of stress agents [68,69] have been employed to bypass the issue of neuronal maturation, but evidence is still lacking far behind to support a molecular and functional resemblance of these cells to adult neurons. Also, it remains unknown whether cellular maturation and ageing are distinct events, and whether and how interfering with one process might affect the other and result in ‘true’ disease-associated phenotypes.

For neurodevelopmental disorders the use of fetal-like neurons for disease modelling could be seen as an opportunity to validate the hypothesis that synaptic formation and establishment during the earliest stages of development are affected. In the case of neurodegenerative diseases the use of these systems to reveal ‘true cellular phenotypes’ remains a significant controversy in the field. On one hand, a significant number of studies have managed to link defects in synaptic connectivity, electrophysiological recordings and synaptic transcriptomes to PD [70,71], AD [72] and HD [73] but data comes mainly using hiPSC-derived systems from familial cases due to dominant mutations rather than sporadic cases that appear later in life. On the other hand, the fact that disease-associated alterations can already be depicted in fetal-like neurons could suggest that synaptopathy starts early, long before clinical symptoms appear and accumulated network miscommunication defects are responsible for the later-appearing phenotypes.

Another major drawback of the 2D neuronal cultures is that they lack the cytoarchitecture of brain tissue, and studies investigating neuronal connectivity defects might be influenced by variations in cell–cell contacts and interactions due to culturing conditions. Limited studies using hiPSC-derived glial cells have revealed a central role in Down's syndrome [74] and SZC pathology [75], and it could be that the presence of multiple CNS cell types will prove necessary to recapitulate more faithfully disease phenotypes. Such an approach has been instrumental to uncover the critical role of astrocytes in the development of neuronal pathology in amyotrophic lateral sclerosis (ALS). In a pioneer study [76] the investigators used human-based astrocyte–neuron co-cultures to demonstrate that astrocytes are critical components in motor neuron degeneration, but more work in different disease models is required to appreciate the contribution of the different CNS cell types in neuronal pathology.

### 4.3. 3D hiPSC-based systems modelling neurological diseases

More recently, three-dimensional hiPSC-based culture systems (table 1), the so-called organoids, have been used to study cell–cell interactions in a context that mimics more closely human development and physiology [88]. hiPSC-derived organoids appear to recapitulate the brain's 3D cytoarchitectural arrangement, thus providing new opportunities to explore disease pathogenesis when derived from patient cells. Organoids can be region-specific, in which case their generation is guided by extrinsic morphogenes and patterning growth factors, yielding forebrain [84], cortical [77], midbrain [78] or hypothalamic structures [82]. Alternatively, organoids can be self-organizing entities, with their assembly relying on intrinsic mechanisms of self-organization [3,88]. Whether region-specific or self-organizing, organoids comprise multiple neural and glial identities, and have the potential to reproduce an anatomically relevant human-specific spatial organization with more complex cytoarchitecture, synaptic connections, cell–cell and cell–extracellular matrix interactions.

**Table 1.** Modelling neurodevelopment and neurological disease using 3D human-based iPSC systems (organoids).

organoid identity	disorder	mutation	phenotypes described	reference
*neurodevelopment*				
cortical spheroids	n.a.	n.a.	functional maturation, synaptogenesis and astrogenesis	Pasca *et al*. [77]
midbrain-like organoids	n.a.	n.a.	functional dopaminergic and neuromelanin-producing neurons	Jo *et al*. [78]
brain microphysiological system	n.a.	n.a.	synaptogenesis; neuron-to-neuron and neuronal-glial interactions (myelination)	Pamies *et al*. [79]
*neurological diseases*				
cerebral organoids	microcephaly	CDK5RAP2 truncating mutations	premature neurogenic non-proliferative divisions	Lancaster *et al*. [80]
telencephalic organoids	idiopathic ASD	n.a.	accelerated cell cycle and overproduction of GABAergic inhibitory neurons	Mariani *et al*. [81]
cerebral organoids	Zika virus exposure	n.a.	decreased neuronal cell-layer volume resembling microcephaly	Qian *et al*. [82]; Yoon *et al*. [83]
forebrain spheroids	Timothy syndrome	CaV1.2 (G406R)	aberrant interneuron migration	Birey *et al*. [84]
cortical organoids	Alzheimer's disease (AD)	APP duplication; PSEN1 M146I; PSEN1 A264E	amyloid aggregation; hyperphosphorylated tau protein; endosome abnormalities	Raja *et al*. [85]
neuroectodermal spheres	Parkinson's disease (PD)	LRRK2 (G2019S)	distinct expression profiles of genes associated with synaptic transmission; synaptic vesicle trafficking	Son *et al*. [86]
cerebral organoids	Huntington's disease (HD)	HTT (60; 109 CAG repeats)	impaired cortical fate differentiation and proper cell organization; immature transcriptional blueprint	Conforti *et al*. [87]

The considerable evolutionary increase in size and complexity of the human brain as compared with other mammalian species, particularly cortical expansion, has been attributed to a greater number and prolonged proliferative potential of neural progenitor cells during development. As hiPSC-derived cortical organoids correspond to human mid-fetal development, they represent suitable models for investigating alterations in individuals with neurodevelopmental disorders [89]. Organoids have been used in the study of lissencephaly, a genetic neurological disorder associated with mental retardation and intractable epilepsy, and revealed neurodevelopmental disease phenotypes and a mitotic defect in outer radial glia, a cell type that is particularly important for human cortical development [90]. Similarly, human forebrain organoids were used to study congenital microcephaly [80] or microcephaly resulting from Zika virus infection of neural precursor cells [83], and more recently ASD [81]. As evidenced from the above paradigms, 3D organoid modelling of neurodevelopmental diseases is still in its infancy, while advances in 3D modelling of neurodegenerative diseases are lacking far behind [85,87].

Even though the 3D cultures present as ideal systems to study the formation and activity of neuronal networks, only two studies published provide relevant in depth information. Detailed electrophysiological analyses of midbrain-like organoid-derived slices revealed APs, spontaneous excitatory and inhibitory post-synaptic currents and large-amplitude excitatory post-synaptic potentials indicative of participation of dopaminergic neurons in network activity [78]. In combination with expression of functional DA receptors, the authors support the potential utility of these systems to evaluate degree of synaptic competence and connections, however it remains to validate the system using hiPSC lines from PD patients. Interestingly, a human 3D brain microphysiological system has been recently developed [79] comprising differentiated mature neurons and glial cells, both astrocytes and oligodendrocytes, that reproduce neuronal-glial interactions and exhibit spontaneous electrical activity as measured by multi-electrode array (MEA), indicative of overall neuronal functionality of the system.

As the 3D systems are still at the early stages of development, complementary use of novel technologies such as 3D printing technologies [82] is expected to improve the scalability and reproducibility of 3D systems, making this approach even more attractive for studying disease pathogenesis and discovering new drugs.

## 5. hiPSC-based models of neurodevelopmental diseases

Since the advent of the cell reprogramming technology, a significant number of hiPSC lines have been generated for diverse neurodevelopmental disorders including monogenic disorders such Rett syndrome, fragile X syndrome and Timothy syndrome, and the more complex pathologies of ASD and SCZ [91] (table 2).

**Table 2.** List of reports on modelling ASD and SCZ using 2D human iPSC-based systems.

disorder	mutation	phenotypes described	reference
*autism spectrum disorders*			
Rett syndrome (RTT)	MeCP2 (various mutations)	reduced dendritic spine density; altered electrophysiological properties; smaller soma size; alterations in Ca^2+^ influx; fewer synapses	Marchetto *et al*. [92]
Phelan–McDermid syndrome (PMDS)	deletions of approximately 1 Mb in chromosome 22	defects in excitatory, but not inhibitory synaptic transmission	Shcheglovitov *et al*. [93]
fragile X syndrome (FXS)	FMR1 (CGG repeat lengths >200)	aberrant neural differentiation	Sheridan *et al*. [94]
FXS	FMR1 (CGG repeat lengths >435)	neurite outgrowth defects	Doers *et al*. [95]
FXS	FMR1 (236 CGG repeats)	impaired neuronal differentiation and function	Lu *et al*. [96]
FXS	FMR1 (150, 250 and 210 repeats)	aberrant neurogenic phenotypes	Boland *et al*. [97]
Timothy syndrome (TS)	CaV1.2 (c.1216G>A)	dendritic retraction	Krey *et al*. [98]; Tian *et al*. [99]
non-syndromic ASD	TRPC6 (t(3;11)(p21;q2 2))	reduction in axonal length and dendritic arborization	Griesli-Oliveira *et al*. [100]
*schizophrenia*			
schizophrenia (SCZ)	various copy number variants (CNVs)	diminished neuronal connectivity; decreased neurite number	Brennand *et al*. [101]
SCZ	various copy number variants (CNVs)	perturbations in cell adhesion molecules in neural progenitor stage	Brennand *et al*. [66]
SCZ	DISC1 (4 bp frameshift deletion)	altered neuronal morphology; glutamatergic synapse defects	Wen *et al*. [102]
SCZ	15q11.2del	deficits in adherens junctions and apical polarity in iPSC-derived neural progenitors	Yoon *et al*. [103]

In this section we discuss studies that provide evidence for synaptic dysfunction either as a result of aberrant neurogenesis or as a failure of synapse maintenance and maturation. Despite the different disorders, the different hiPSC origins within the same disease, the various differentiation protocols, culture systems and experimental approaches, some common themes emerge from these studies. A number of genes affecting early neuronal development and newly identified phenotypes in early progenitors suggest that shared mechanisms must operate in the initiation of these diseases. Finally, for ASD and SCZ, which have complex genetics with over one hundred disease-risk alleles, each having modest effects, the fact that hiPSCs and their derived differentiation products have exactly the same genetic make-up as the patient, which is impossible to replicate in experimental animal models, highlights once more the power of these models.

### 5.1. Autism spectrum disorders

Autism spectrum disorders (ASD) are prototype neurodevelopmental pathologies that include Fragile X syndrome (FXS), Rett syndrome (RTT), and William's syndrome. It is estimated that ASD affects approximately 1 in 70 children that exhibit early onset symptoms persisting throughout life and producing significant impairments in social, communicative, cognitive and behavioural functioning [104]. ASD individuals manifest restrictive repetitive and stereotyped behaviour and interests, and often have seizures and intellectual disability. The aetiology is still unclear with both genetic and environmental factors being involved in ASD pathogenesis. Genome-wide association studies (GWAS) have uncovered a large number of mutations and/or polymorphisms in genes encoding for proteins that affect transcriptional control, chromatin remodelling, protein synthesis, cellular metabolism, development and function of synapses [105]. A consistent feature in neurons of these patients is an abnormal dendritic structure and alterations in spine morphology [106–108]. These defects have also been observed in mouse models for ID and ASD [109,110]. Most of the ID/ASD related proteins have been shown to play essential roles affecting dendritic spine structure and number, eventually leading to altered neuronal connectivity [111]. Around 600 genetic variations (SFARI database) affect synaptic genes [112] and the imbalance between excitation and inhibition in neocortical areas is a key feature underlying ASD pathogenesis. Multiple rare genetic variants in synaptic proteins implicate defects in synaptic adhesion pathways [113]. Other ASD-associated genes are involved in activity-dependent synapse elimination, a process that defines neuronal circuit plasticity throughout life. Using whole-exome sequencing to analyse 15 480 DNA samples, a number of rare *de novo* single nucleotide variants (SNVs) in ASD were identified in genes whose protein products are involved in synaptogenesis and dendritic morphogenesis, including the sodium channel SCN2A, E3 Ubiquitin ligases, miR-137 and multiple PSD-associated proteins [114]. Based on these genetic profiles and complementary animal-based studies, it seems that ASD pathology results directly from defects in synapse organizing molecules.

Keeping in mind that ASD features appear at the time of brain development when sensory experience modifies excitatory synapse maturation or elimination and promotes inhibitory synapses, it is not surprising—though still remarkable—that ASD hiPSC-derived neurons show decreased synaptic connectivity *in vitro*, morphologically immature synapses and decreased neuronal activity as demonstrated by lower spontaneous and evoked post synaptic currents [108]. The first study to develop a hiPSC model for ASD was based on a case of RTT, a syndromic type of ASD that is linked to a mutation in the MECP gene on X chromosome [92]. Neurons displayed reduced dendritic spine density, altered electrophysiological properties, smaller soma size, alterations in Ca^2+^ influx and fewer synapses, which could be partially rescued by insulin growth factor 1 (IGF1). In a later study using hiPSCs from ASD patients with SHANK3 deletions [93], which are also associated with ID and SCZ, derived neurons had major defects in excitatory, but not inhibitory synaptic transmission. In this study, the authors stress out that their findings differed significantly from the phenotype observed in a relevant mouse model and highlight the importance of using closer-to-human models to understand disease pathogenesis.

Interestingly, a number of mutations associated with autism are located in genes encoding proteins involved in the process of initiation and propagation of electrical signals, including calcium channels [115]. Specifically, mutations in the CACNA1C gene cause an abnormal function of this calcium channel, and have been associated with BP, SCZ and another syndromic form of ASD, Timothy syndrome (TS). hiPSC-derived neurons from TS patients presented dendritic retraction [98] and a similar observation was made in hiPSC-derived neurons carrying a mutation in another Ca^2+^ channel, the Cav1.2 [99]. Even though information from non-syndromic cases of ASD is more difficult to reproduce and validate, these studies strongly support that the core of ASD pathology lies at the synapse. An individual carrying a *de novo* inherited genetic variation in the TRPC6 gene, which encodes an important protein controlling neuronal function [100], was used to derive mutant neurons that exhibited profound reduction in axonal length and dendritic arborization that was also partially improved by IGF1 treatment, further underlying the importance of using syndromic cases of ASD to reveal important pathways for autism.

In another ASD syndromic type, FXS, which remains the most common type of inherited ID, neurons derived from hiPSCs of affected individuals showed aberrant neural differentiation and impaired neuritic initiation and outgrowth with corresponding altered gene expression of transcripts associated with synaptic structure and activity [94,95]. Using hiPSC-derived neurons with a corrected version of this genetic area (through gene complementation), this study confirmed that the FXS phenotype is due to an expansion of CGG repeats in the 5′ UTR of the FMR1 gene on the X chromosome. FXS neurons also displayed alterations in pre- and post-synaptic protein levels, including SHANK3, and defective Ca^2+^ influx [95,116]. Two recent studies [96,97] have used RNA-Seq to identify transcriptional misregulation in FXS-hiPSCs during neuronal differentiation and showed a clear aberrant neurogenic profile in genes associated with developmental signalling (WNT and BMP pathway), adhesion (Cadherins, SLITRKs) and maturation, while a large number of identified genes were associated with ASD (FXS candidate genes compared with the curated list of ASD-associated genes from SFARI). Overall the FXS-based studies provide strong evidence for synaptic dysfunction due to neurodevelopmental delay.

Even though the genetic architecture of ASD is highly complex and novel variants are identified continuously that await to be established as causative in hiPSC models, the data collected so far clearly indicate both abnormal neurogenesis and synaptogenesis, resulting in defective neuronal networks (table 2). Additional evidence for aberrant neurogenesis in ASD has come from the detailed molecular and structural characterization of hiPSC-derived organoids from idiopathic ASD patients with no known underlying genomic mutation. Transcriptomic and morphometric cellular analyses have revealed an accelerated cell cycle of progenitor cells and overproduction of GABAergic inhibitory neurons caused by increased FOXG1 gene expression [81]. Unlike previous findings indicating a deficiency in synaptic connections in ASD individuals carrying loss-of-function mutations in synaptic adhesion molecules (SHANK, NLGN, NRXN), transcriptomic analysis of patient-derived organoids revealed upregulation of mRNAs for the synaptic adhesion molecules NLGN1, NRXN1, NRXN2 and NRXN3, and exuberant synaptic development. This is in line with a gain-of-function mutation in NLGN3 found in ASD patients that has been shown to confer an increase in GABAergic synaptic signalling [117–119]. It therefore seems that the balance, rather than the absolute numbers, of glutamate (excitatory) and GABAegric (inhibitory) neurons is important for proper functional outcome [81].

### 5.2. Schizophrenia

Synaptic dysfunction has also been identified at the core of SCZ pathology. SCZ is a major neuropsychiatric disorder affecting around 1% of the population worldwide. Affected individuals manifest positive (delusions and hallucinations) and negative symptoms (lack of volition and blunted effect) accompanied by mood, cognitive and motor dysfunction [120].

Family studies show that SCZ is predominantly a genetic disorder with around 80% of heritability risk. GWAS screens, family studies and exome sequencing analyses have demonstrated that mutations are enriched in genes involved in neuronal excitability and plasticity, including numerous synaptic genes and calcium channels (NRGN, CACNA1C, CACNB2) [64,121–123]. One of the high-risk SNVs is an exonic deletion in the neurexin1 gene (NRXN1), encoding a cell adhesion molecule known to regulate the formation and maintenance of synapses and determine the pre-synaptic organization [124]. Even though a deficit in cognition is recognized as a central feature in SCZ, the picture so far is not as clear as in the case of ASD, and a causal relationship between synaptic protein defects and SCZ pathophysiology remains an open question.

Despite the fact that SCZ is a pathology that manifests late in adolescence or early in adulthood, pathological features such as loss of grey matter and reduced number of synaptic structures, and impairments in higher functions such as cognition, perception and motivation, propose defects at the synapse level that could be initiated early in development. In support of this hypothesis is: (i) a number of damaging de novo mutations in genes co-expressed in the prefrontal cortex during development; (ii) the strong SCZ genetic association to genetic markers across the MHC locus; and (iii) the genome variation in complement component 4 (C4) gene [125]. Both human MHCI and C4 are localized specifically in neuronal synapses, dendrites, axons and cell bodies, and experiments in rodents have revealed their essential role in synapse elimination during early postnatal development [126]. A number of hiPSC models have been developed since the first proof-of-principle study of Brennand *et al.* in 2011 [101], where they reported that neurons derived from four patients differed in neuronal connectivity, morphology and gene expression when compared to control cells (table 2). Even though the authors did not identify defects in synaptic function despite the observed decrease in synaptic connections and the reduced expression of transcripts involved in axonal growth and synaptogenesis (SLIT/ROBO, EFNA, cell adhesion molecules), they attributed it to limitations of the assays used. The same group four years later [66] looked for potential alterations in SZC hiPSC-derived neuronal progenitors (NPCs) based on the rationale that hiPSC-derived neural cells maintained for 6 weeks in culture share similarities with those in first-trimester fetal brain tissue. Proteomics analysis at the progenitor stage identified perturbations in cell adhesion molecules (NCAM, cofilins CFL1 and 2, NLGN3), while SCZ neurospheres showed aberrant migration *in vitro*, providing evidence for early initiating events in SZC. These studies were based on heterogeneous cohorts of SCZ patients, without prior knowledge of their genetic risk variants. In another study, mutant neurons from hiPSCs carrying the 15q11.2 microdeletion [103], reported to be a risk factor for SCZ [127], had significant defects in neural rosette formation with disrupted adherens junctions and scattered expression of atypical PKC*λ*, a marker of apical polarity.

On the other hand, data showing defective neuronal networks forming in SZC came from the study of Wen *et al.*, which took a genetics oriented approach by analysing the effect of DISC1 mutations that co-segregate with major psychiatric disorders. hiPSC lines from two patients carrying the same frame shift mutation in DISC1 were used for neuronal differentiation [102]. Data revealed altered neuronal morphology, accompanied by problematic electrophysiological recordings indicative of glutamatergic synapse defects. In this study not only was the role of DISC1 in SCZ confirmed, but the causal role of DISC1 in regulating synapse formation was verified as experiments were performed in isogenic control lines. Interestingly, the mRNA of presynaptic proteins (SYN2 and 3, SYP, SYNPR, NRXN1 and VAMP2) was increased in mutant neurons but the post synaptic GLUR1 and GRIN1 were not altered, suggesting that SCZ might be primarily due to presynaptic defects.

## 6. hiPSC-based models of neurodegenerative diseases

Even though synaptopathy is a core issue in neurodegenerative diseases, it is generally believed that it is the consequence rather than the initiating event of an on-going degenerative process at disease-relevant brain areas. Thus the progressive loss of nigrostriatal neurons in PD, of striatal medium spiny neurons in HD and of cortical neurons in AD are ultimately thought to result in weakening and loss of synaptic integrity in these pathologies, thus conferring cognitive deficiencies [33,128,129]. However, a number of familial cases of PD are causally related to inherited mutations in synaptic genes, such as α-synuclein [31], and HD is due to a CAG repeat expansion in HTT [130]. In addition, significant cognitive symptoms in neurodegenerative diseases appear early and subtle differences in higher functions are identified in the pre-symptomatic phase.

Below we focus on findings from hiPSC-based models of HD and PD that exhibit synaptopathy features and have aided us to gain more insight into the neurodevelopmental component of HD and the overall neuronal network dysfunction in PD.

### 6.1. Huntington's disease

Huntington's disease (HD) is characterized by motor abnormalities, psychiatric symptoms and cognitive deficits. It is caused by a CAG repeat expansion in the HTT gene encoding a polyglutamine tract expansion in the HTT protein [130]. CAG repeats of 40 or greater cause adult HD, while greater than 60 cause juvenile HD, suggesting that the extent of repeats is directly correlated with age of clinical onset. Neuropathology confirms that cortical atrophy and loss of striatal medium spiny neurons are the hallmark of the disease. Additionally, adult neurogenesis seems to be impaired in the striata of HD patients [131] with increased cell proliferation and absence of adult born neurons [132]. Neuroimaging scans of pre-manifest HD-affected brains detected changes in striatal, cortical and whole brain volume before symptoms appear [133,134]. Within the framework of the extensive longitudinal research study PREDICT-HD, HD patients were followed for decades before symptoms became apparent and we now know that HD patients develop clinical changes years before diagnosis can be made [135]. These include cognitive, psychiatric and functional changes, together with altered brain morphology and connectivity, as shown in fMRI. Importantly, children at risk of HD exhibit smaller head size [136], suggestive of neurodevelopmental abnormalities.

hiPSC studies have provided data for unexpected disturbances in early developmental processes in classical neurodegenerative diseases, such as HD (table 3). Even though clinical evidence from pre-symptomatic individuals or subjects with prodromal symptoms pointed to neurodevelopmental abnormalities, the late onset of serious motor and cognitive dysfunction associated with HD and lack of suitable model systems led us to neglect the dispersant evidence until recently, when the early processes relevant to developmental defects were recognized [152]. A number of studies indicate that HTT is necessary for brain development [153–155]. An important first study showed that mouse embryonic stem cells lacking HTT are unable to from *in vitro* neural rosettes, which correspond to the presumptive neuroepithelium [156]. Moreover, conditional silencing of HTT in the developing mouse cortex revealed that this protein is required for correct establishment of cortical and striatal excitatory circuits, a function that is lost when mutant HTT is present [157]. Further, striatal neural progenitors are compromised in HD patients and relevant mouse models [158].

**Table 3.** List of reports on modelling HD and PD using 2D human iPSC-based systems.

disorder	mutation	phenotypes described	reference
*Huntington's disease*			
Huntington's disease (HD)	HTT (42/44; 39/42; 17/45 CAG repeats)	increased lysosomal activity	Camnasio *et al*. [137]
HD	HTT (60; 109 CAG repeats)	altered gene expression of neurodevelopmental pathways and synaptic homeostasis	HD iPSC Consortium 2017 [73]
*Parkinson's disease*			
Parkinson's disease (PD)	LRRK2 (G2019S)	increased susceptibility to oxidative stress	Nguyen *et al*. [68]
PD	LRRK2 (G2019S)	increased susceptibility to proteasomal stress	Liu *et al*. [138]
PD	LRRK2 (G2019S)	increased susceptibility to oxidative and mitochondrial stress; diminished neurite outgrowth	Reinhardt *et al*. [139]
PD	GBA (RecNcil; L444P; N370S)	autophagic/lysosomal deficiency; impaired Ca^2+^ homeostasis	Schondorf *et al*. [140]
PD	GBA (N370S)	DA homeostasis defects	Woodard *et al*. [141]
PD	PARK2 (various mutations)	impaired dopaminergic differentiation; mitochondrial alterations	Shaltouki *et al*. [142]
PD	PARK2: EX3-5DEL; PARK2: EX3D EL	reduced complexity of neuronal processes	Ren *et al*. [143]
PD	PARK7 (c. 192G > C)	mitochondrial and lysosomal dysfunction	Burbulla *et al*. [144]
PD	SNCA (G209A)	n.a.	Soldner *et al*. [145]
PD	SNCA triplication	increased susceptibility to oxidative stress	Byers *et al*. [146]
PD	SNCA (G209A)	increased susceptibility to oxidative and nitrosative stress	Ryan *et al*. [147]
PD	SNCA (G209A); SNCA triplication	increased nitrosative stress; ER stress	Chung *et al*. [148]
PD	SNCA triplication	increased susceptibility to oxidative stress	Flierl *et al*. [149]
PD	SNCA triplication	impaired neuronal differentiation; compromised neurite outgrowth	Oliveira *et al*. [150]
PD	SNCA (G209A)	defective synaptic connectivity; axonal neuropathology; altered expression of synaptic transcripts	Kouroupi *et al*. [70]
PD	SNCA (G209A)	fragmented mitochondria and αSyn deposits at mitochondrial membranes in response to cardiolipin	Ryan *et al*. [151]

Recently, developmental alterations in HD cells have been identified that show that mutant HTT impairs developmental pathways by disrupting synaptic homeostasis and increases vulnerability to the pathogenic consequences of polyglutamine repeats over time. In the elegant study of the HD iPSC Consortium, hiPSC lines from non-diseased individuals (21–33 CAG repeats) and juvenile onset HD patients (60–109 repeats) were differentiated into mixed cultures containing neurons and progenitor cells [73]. RNA-Seq and pathway analysis of differentially expressed genes between patients and unaffected individuals showed alterations, with a notable 59% of transcripts being associated with nervous system development and function. Essential neurogenesis factors (NEUROD1 and GAD1) were downregulated, while axonal guidance, WNT signalling, Ca^+^ signalling (subunits of the voltage gated CACNA1 channel, plasma membrane Ca^+^ ATPase, CAMKII, CALM and CREB), glutamate (NMDA and AMPA receptors, SLC1A3 and SLC1A6) and GABA receptor (GAD1 and GAD2) signalling were markedly dysregulated in HD lines. The authors also performed ChIP-seq analysis and revealed chromatin signatures consistent with impaired cell maturation, while meta-analysis to compare profiles with changes in gene expression during human striatal maturation showed a clear overlap in the core network of genes essential for normal development of the human striatum. This is the first study using juvenile HD-hiPSC lines, and further analysis, including lines derived from adult onset HD that contain less CAG repeats, will be valuable to confirm if CAG number is associated with early defects or is independent and plays a role during disease course only. Nevertheless, this is an important study demonstrating HD-associated neurodevelopmental defects that may disrupt brain homeostasis, establishing a vulnerability to later effects of mutant HTT, as demonstrated in the first study reporting analysis of hiPSC-derived neurons from rare homozygous and heterozygous HD patients [137]. Most important is that it shifts the perception that neurodegenerative diseases start late in life and underlines the importance of assessing patients at risk earlier. Correlating clinical data with hiPSC-derived findings is vital for appreciating the initiating steps in such pathologies.

The first HD organoid-like structure was recently reported by the Cattaneo group [87], and demonstrates that mutant HTT affects the ability of hiPSC to generate cortical and ventral striatal telencephalic identities, not only supporting previous data on the developmental component of HD but providing the precise steps of development affected by the mutant protein.

### 6.2. Parkinson's disease

Parkinson's disease (PD) is the second most common neurodegenerative disorder, classically associated with an extensive loss of dopaminergic neurons of the substantia nigra pars compacta [159,160]. The hallmark of the disease is accumulation of pathogenic conformations of the pre-synaptic protein α-synuclein (αSyn) and the formation of intraneuronal protein aggregate inclusions, termed Lewy bodies or Lewy neurites. Neurodegeneration of dopamine neurons leads to a prominent dopaminergic deficiency in the basal ganglia, responsible for motor disturbances [161]. However, it is now recognized that the disease involves more widespread neuronal dysfunction, leading to early and late non-motor symptoms such as hyposmia, autonomic dysfunction, sleep disturbances, hallucinations, depression, cognitive decline and dementia [162]. These observations have shifted the focus from a defect in dopamine neurons to a more general neuronal disruption, suggesting that the pathological mechanisms lie outside the substantia nigra and may be initiated long before neuronal loss [163,164]. Advances in brain imaging techniques and analysis of post-mortem tissues show extensive Lewy body pathology in other brain areas, while familial PD cases often appear and progress with an absence of motor symptoms [128]. Previous work on post-mortem tissue of another synucleinopathy, namely dementia with Lewy bodies (LBD), demonstrated that the majority of αSyn aggregates are located to pre-synaptic terminals with almost complete loss of dendritic spines at the post-synaptic area, suggesting that synaptopathy is a central event in the initiation of neurodegeneration in LBD [165] and probably of PD as well.

In a similar approach to HD, rare familial cases of PD are now being followed during the asymptomatic phase [166], before serious motor and cognitive symptoms appear; however, it is still too early to argue for similar-to-HD pre-symptomatic clinical changes.

Nevertheless, evidence coming from animal models of PD supports the hypothesis that pre-synaptic accumulation of αSyn impinges on synaptic function and axonal integrity leading to degeneration and cell death. Overexpression of αSyn in mice inhibits neurotransmitter release, reduces the size of the synaptic vesicle recycling pool and alters the ultrastructure of the nerve terminal before any signs of neurodegeneration are observed [167]. In another study with overexpression of αSyn mutants, decelerated vesicle transport was observed in neurons leading to autophagy and axonal degeneration [168]. Recently, ultrastructural analysis of mouse knockout synapses for all three members of the synuclein family (α, β, γ) demonstrated their direct involvement in controlling synapse size and synaptic vesicle distribution [169]. These disturbances at the synapse level may be detrimental, especially if considering that the dopaminergic neurons lost in late PD stages possess axons containing more than 1 million synapses. Overall these findings underpin the concept that familial cases of PD, and possibly sporadic PD, may be primarily synaptopathies.

Models of familial PD were among the earliest hiPSC-based disease models to be generated since the advent of cellular reprogramming. Even though hiPSCs have been derived from patients with idiopathic PD, the majority of studies have focused on familial PD cases caused by mutations in a single gene (table 3). Although these are rare forms of PD, they provide a clear advantage: the observed phenotypes are attributable to a specific gene alteration and therefore causality may be established. Today, mutations in 14 genes have been identified to cause familial PD [170]. From those, the best known are implicated in both autosomal and recessive forms causing early disease onset with a generally severe clinical phenotype, and include αSyn, leucine-rich repeat kinase 2 (LRKK2), β-glucocerebrosidase (GBA) and various PARKIN genes. Several studies utilizing hiPSC models reported neuronal dysfunction associated with mutations in LRRK2 [68,138,139], GBA [140,141], PARK2 [142,143], PARK7 [144] and αSyn [70,171–173]. Even though in most PD studies the aim has been to generate and characterize dopaminergic neurons, few studies have included other types of neurons in the analysis, including glutamatergic and GABAergic neurons [70,148]. Overall, data derived from these studies confirmed the involvement of various pathways previously implicated in PD pathogenesis, such as mitochondrial, lysosomal and endoplasmic reticulum dysfunction, impaired clearance of autophagosomes, disturbed calcium homeostasis and oxidized dopamine accumulation. However, it has been challenging to identify cellular pathologies in hiPSC-derived PD neurons in the absence of oxidative or other cellular stress.

LRRK2 mutations represent the most common cause of familial PD and are autosomal dominant with age-dependent penetrance [174]. This kinase is highly expressed in brain areas receiving dopamine innervations, such as the striatum, hippocampus, cortex and cerebellum [175], and has been associated with many aspects of neuronal function including neurogenesis, axonal outgrowth and synaptic function [176–179]. Mice carrying the LRRK2-G2019S mutation have increased basal synaptic efficiency and reduced long-term depression not associated with presynaptic changes, but probably due to enhanced AMPAR-mediated synaptic transmission [180]. This pre-clinical data was not confirmed in the initial studies that generated hiPSC-derived neurons from LRRK2-G2019S patients. Dopaminergic neurons were particularly susceptible to oxidative and mitochondrial stress [68,138,139], but RNA-sequencing analysis did not reveal changes in transcript expression associated with synapse formation and function [139]. The only impairment relevant to synapse formation was a diminished neurite outgrowth velocity, a phenomenon not specific to dopamine neurons [139]. Despite the fact that these LRRK2-G2019S mutant neurons also had increased levels of αSyn and TAU proteins, a phenotype previously associated with axonal degeneration and synaptic alterations, neuropathology was not observed. As this mutation has an age-dependent appearance of clinical phenotype, it could be that the end time point of analysis was too early to reveal such defects. In a similar way, the hiPSC-GBA1 mutation PD systems did not provide a link to dysregulated synaptogenesis or synaptic function. Since the studies performed are quite limited in number and the focus of the initial analysis might not have been to depict differences in synaptic function, additional work is required to draw safe conclusions about the presence of synaptopathy in LRRK2 and GBA mutant neurons.

In contrast, αSyn hiPSC-based systems have been far more informative in providing clues for early synaptic deficits in pathology initiation and progression. Even though mutations in αSyn account for a small number of familial PD cases, they have received particular attention and have been employed extensively by researchers to create both animal models [181] and hiPSC-based cellular platforms of neurons and progenitor cells [173]. The reason is that the first genetic cause of PD to be identified was the G209A mutation in the αSyn gene SNCA, leading to synthesis of the pathological p.A53T-αSyn mutant protein [182]. αSyn protein was soon after discovered to be the major component of Lewy bodies, the pathological hallmark of both familial and sporadic PD [183]. Since then a number of point αSyn mutations have been identified—A30P, E46 K, H50Q, G51D and A53E [184–189], as well as duplication or triplication of the αSyn gene locus which also causes dominant and severe forms of PD [170,190].

In a first study by Jaenisch and colleagues [145], successful derivation of hiPSC-derived p.A53T and p.E46 K lines and isogenic gene corrected controls were reported, without further characterization. This p.A53T-hiPSC line was used in a later study by Ryan *et al.* [147] to produce cultures of midbrain dopamine neurons that displayed aggregated αSyn 35 days after differentiation in both the cell soma and neurites, features similar to those previously identified in post-mortem brains from p.A53T patients [191,192]. Despite the presence of αSyn oligomeric aggregates, dopamine neurons did not show axonal damage or defective neuronal network formation. In a more recent study the p.A53T neurons displayed fragmented mitochondria and αSyn deposits at mitochondrial membranes in response to cardiolipin, a mitochondrial membrane lipid [151]. Cortical neurons generated from the same set of p.A53T hiPSC lines by Lindquist and colleagues were also susceptible to induced ER stress [148]. These studies support a ‘two-hit’ hypothesis where the mutant background facilitates induction of a PD phenotype by environmental toxins.

The first observation of damaged neurites and axonal fragmentation with multi-electrode arrays revealing asynchronous firing and a reduction in the number of active channels was identified in LRRK2 neurons. RNA-sequencing data from all PD lines showed a consistent upregulation of the RNA-binding protein fox-1 homologue (RBFOX1), a neuron specific factor that regulates neuronal splicing networks and controls neuronal excitation [193]. Interestingly, when the authors mapped significant differential splicing products they generated a list of 41 genes with a profound enrichment in GO terms related to neuron projection and neuronal activity. From those, they confirmed that GRIN1 (an NMDA receptor subunit) and SNAP25 (a key component of the SNARE complex) specific isoforms were altered in PD neurons, demonstrating for the first time that differential splicing events are regulated by RBFOX1 in these cellular systems.

Recently, a study from our group [70] further enhanced the hypothesis that synaptopathy is an early event in familial PD cases. This work was focused on the analysis of newly generated lines from two p.A53T patients with different clinical progression and severity [70]. At 35 days of differentiation to dopaminergic neurons following a dual SMAD inhibition protocol [49,194], cells exhibited clear features of neurodegeneration, including extensive neuritic pathology, αSyn^+^ and Tau^+^ swollen varicosities and large spheroid inclusions highly similar to the dystrophic neurites identified in the brain of p.A53T patients [192,195]. Astonishingly, the severity of the cellular phenotype was directly correlated with the clinical picture of the two different patients. In a similar manner to the observations of Ryan *et al.* [147], thioflavin-positive aggregates started to be visible at 35 days of differentiation while they became more prominent and widespread at 50 days, with αSyn protein also being co-detected. A connection of the degenerative phenotype to αSyn pathology was established in our study, since small molecules inhibiting αSyn aggregation reverted the neurodegenerative phenotype, indicating a potential treatment strategy for PD and other related disorders.

An intriguing observation was that the extensive p.A53T pathology appeared without the need for external neurotoxic or oxidative stress. Axonal degeneration was evident in dopaminergic, but also in glutamatergic and GABAergic, neurons present in our culture system, as well as in betaIII-tubulin-positive neurons prior to subtype specification. We presume that the simultaneous presence of all three major neuronal subtypes might be the key for the strong intrinsic and widespread p.A53T degenerative phenotype. This notion is also supported by the observation that when using the Kriks *et al.* [196] differentiation protocol to enrich for dopamine neurons, the fraction of GABAergic cells is clearly diminished and the p.A53T-related axonal degeneration is also less noticeable (E.T., G.K., O.Z. & R.M. 2018, unpublished data). Even though we cannot exclude the possibility that different patient lines may yield neurons with variable phenotypic characteristics, it is also likely that differences in the ratio of excitatory to inhibitory neurons within a culture may be a decisive factor for the phenotypic outcome.

Transcriptional profiling of p.A53T neurons in Kouroupi *et al.* [70] also revealed dysregulated molecular pathways in the absence of external stress conditions. Pre-synaptic vesicle formation and trafficking molecules (SYN3, SV2C, RPHA3, DOC2B), vesicular and plasma membrane neurotransmitter transporters, synaptic cell adhesion (SLITRKs, Cadherins) and post-synaptic density (DLGAP2, GRIN2D, GRIP2)-associated mRNAs were all decreased in the p.A53T neurons. This correlated well with compromised neuritic growth and defective synaptic connectivity. Notably, both axonal guidance molecules and WNT family members associated with synaptogenesis were significantly altered, suggesting perturbations during synaptogenesis. From our data we cannot infer defects at a specific part of the synapse in p.A53T pathology, and ultrastructural analysis is needed for such correlations. However, considering the localization of αSyn at the pre-synaptic area and previous observations from overexpression studies in animal models where ‘vacant synapses’ were formed [197], it could be that an original misorganization of the pre-synaptic area might affect the overall organization of the trans- and post-synaptic sites. As the most striking mis-expression was noted in trans-synaptic adhesion molecules, we could also assume that correct alignment for proper synaptogenesis and maturation could not be achieved, further affecting the pre- and post-synaptic regions.

Although this remains an open question, our study clearly indicates synaptopathy as a major feature in p.A53T pathology that is initiated early.

Evidence for aberrant neurogenesis in PD has come from the analysis of hiPSC derived neuronal progenitors (NPCs) and neurons from PD patients harbouring a triplication of the αSyn locus. These progenitors demonstrated relevant susceptibilities to oxidative [146,147] and nitrosative stress [148], and had reduced capacity to differentiate into dopaminergic or GABAergic neurons, while they displayed compromised neurite outgrowth and lower neuronal activity as compared with control cultures [150]. This is the first report to show a link between αSyn and developmental processes in hiPSC-derived cell systems. Molecular profiling indicated lower levels of differentiation markers such as TH, NURR1, GABABR2 and DLK, but also lower GIRK2, consistent with the lower potassium currents observed. Even though isogenic control lines were not included in this experimental setting and someone could argue that this effect is not αSyn dependent as the triplication of the locus affects the expression of 3 up to 12 genes, knocking down αSyn with a lentivirus rescued the differentiation defects in one out of the two lines used. Nevertheless, such differentiation distortions were not reported in a follow-up study using a different set of triplication lines [71], probably due to the clonal variation and differentiation propensity of the lines generated. This follow-up study [71] also included a Parkin and six LRRK2 mutant lines, and despite the neurite outgrowth defects observed in midbrain dopaminergic neurons, the number of TH^+^ neurons was unaffected.

Until now, the sole 3D model for PD has been generated from patient hiPSCs carrying the G2019S mutation in LRRK2 [186]. In this work, the investigators allowed mutant hiPSCs to differentiate into 3D human neuroectodermal spheres and performed microarray analysis that revealed altered mRNA levels in synaptic vesicle trafficking molecules, including Synapsin 2 and 3. However, neuronal activity recordings were not performed, limiting the capacity to draw safe conclusions for synaptic defects in this system.

## 7. New perspectives for understanding synaptic dysfunction in neurological disease

Molecular and cellular analysis of hiPSC-derived CNS cell subtypes from ASD, SCZ, HD and PD (both sporadic and familial cases) has confirmed that ASD and SCZ have a strong developmental component, has yielded valuable information on early disease events and has identified unexpected early neuronal disturbances in PD and HD. However, it remains unknown if the initiation of these pathologies involves aberrant neurogenesis and synaptogenesis or is the result of late-appearing defects in healthy synapses, or even both. The latter has been validated using HD hiPSC-based models that helped dissecting the role of HTT both in aberrant neurogenesis [73] and neuronal degeneration [198]. As more hiPSC lines are generated by implementation of diverse differentiation procedures, and novel tools are developed to monitor neuronal function both at the single-cell and the network level in a highly controlled temporal manner, future studies hold great promise in identifying initiating disease events.

Protein aggregation in neurodegenerative diseases has been considered a central cause of neuronal dysfunction due to perturbations in proteostasis [36,37]. The detection of aggregates has not been consistently achieved in hiPSC-based models of PD and HD, and this is usually attributed to intrinsic clonal differences and alternative differentiation procedures while in some cases it is facilitated by the addition of cellular stressors. Until now studies in hiPSC models have not addressed whether protein aggregation and synaptic defects are interrelated processes, and future studies are expected to shed light on the spatio-temporal events of these mechanisms.

Although the causality of neurodevelopmental and neurodegenerative pathologies has been addressed separately, emerging evidence reveals commonalities that can no longer be overlooked. Hollander *et al.* suggested links between ASD and PD based on the overlapping phenomenon of repetitive behaviours, with a common underlying involvement of the basal ganglia [199]. Motor deficits have also been reported in ASD infants prior to the typical time of ASD diagnosis [200]. In 2015, during a study that aimed to examine ASD in older subjects (more than 50 years old), high rates of Parkinsonism were observed [201]. To follow up this observation, a second group of adult ASD patients was assessed systematically and a remarkable 20% of those manifested signs of Parkinsonism [201]. In addition the genetic link of Asperger syndrome with Parkin2, a classical juvenile PD-associated gene, also suggests a possible overlap [202]. At the same time, alterations of dopamine metabolism in neurodevelopmental disorders is no longer a hypothesis, but is well supported by a wealth of evidence derived from both neuroimaging and genetic studies. Specifically, in SCZ patients, there is increased dopamine uptake and storage in the basal ganglia, while, large-scale genetic studies show an association with gene loci coding for dopamine receptors, as well as for several proteins mediating synaptic transmission [203].

Identifying possible converging mechanisms of neurological diseases will be aided by the detection of disease hallmarks and features of one neurological disease, in hiPSC-derived cells from another pathology. A characteristic example is the case of Down's syndrome, a developmental disorder that, apart from intellectual disability and epilepsy, is characterized by early-onset Alzheimer's [204]. hiPSC-based models have clearly shown impaired neurogenesis and abnormal dendritic and synaptic morphology [205,206], but most importantly have been used to link the two pathologies by showing that Down's syndrome cortical neurons secreted pathogenic Aβ peptide and their cell bodies and dendrites contained hyperphosphorylated Tau protein, a hallmark of AD neurons [45]. The meta-analysis of the p.A53T-neuron molecular profile performed by our group [70], where differentially expressed synaptic transcripts were investigated for their association with other neurological disorders, also provides a good starting point to consider disease overlapping mechanisms. Apart from the expected overlap with PD and AD, we found a significant number of genes associated with ASD, SCZ and bipolar disorder. Notably, these included transcripts for adhesion proteins such as CDH13, CDH9, CDH15 and SLITRKs, all strongly linked to ASD [207,208], presynaptic molecules such as SYNIII and SV2C, associated with non-syndromic autism and SCZ [209,210], and post-synaptic transcripts such as DLGAP2 and GRIN2D, linked to SCZ [211–213]. Such approaches are only at the beginning and are expected to yield valuable information on overlapping and probably converging disease features that could support drug repositioning strategies.

**Figure 2.**
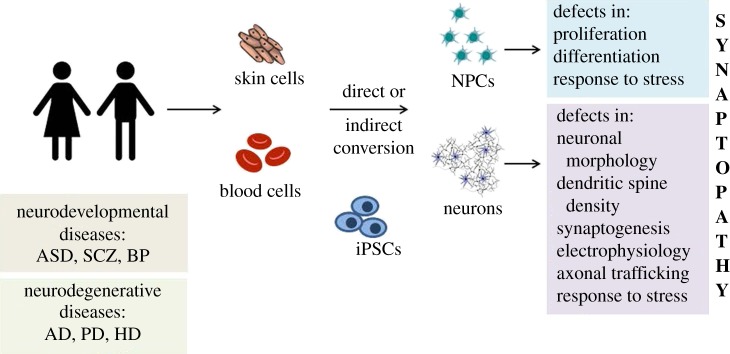
Scheme of neurological disease modelling using cell reprogramming technology. ASD, autism spectrum disorders; SCZ, schizophrenia; BP, bipolar disorder; AD, Alzheimer's disease; PD, Parkinson's disease; HD, Huntington's disease; iPSCs, induced pluripotent stem cells; NPCs, neural progenitor cells.

Until 2007, improved disease modelling was largely dependent on the generation of novel animal models in an effort to recapitulate more faithfully human pathology. A decade later, disease modelling is performed in a human setting and in a patient-specific manner using hiPSC-based systems (figure 2) that not only enable us to study diseases but also human neurodevelopment and neurophysiology. Despite limitations arising from patient variability, experimental design and data interpretation, the use of hiPSCs has revolutionized the way we define neurological diseases. New links between disease phenotypes, gene and protein expression profiles, and cellular responses to drugs are now feasible, while overlapping characteristics have been revealed in pathologies previously considered non-related. Collecting data from a number of hiPSC models from a variety of such diseases reveals early synaptic dysfunction as a common feature (figure 2). The developmental aspect of ASD and SCZ has been largely confirmed, while it implies a newly emerging concept for HD and PD that calls for further investigation. Collaborative efforts, standardized methods and sustainable support are essential to address important unresolved questions. Establishment of synaptopathy as a critical event in the pathogenesis of neurodegenerative disorders should create new prospects for treatment strategies. Without doubt hiPSC technology holds great promise for disease modelling and drug discovery, and this potential is now beginning to be realized.

## Data accessibility

This article has no additional data.

## References

[1] Torres VI, Vallejo D, Inestrosa NC 2017 Emerging synaptic molecules as candidates in the etiology of neurological disorders. Neural Plast. 2017, 8081758 (10.1155/2017/8081758) 28331639 PMC5346360

[2] Takahashi K, Tanabe K, Ohnuki M, Narita M, Ichisaka T, Tomoda K, Yamanaka S 2007 Induction of pluripotent stem cells from adult human fibroblasts by defined factors. Cell 131, 861–872. (10.1016/j.cell.2007.11.019) 18035408

[3] Pasca SP 2018 The rise of three-dimensional human brain cultures. Nature 553, 437–445. 29364288 10.1038/nature25032

[4] Wilfert AB, Sulovari A, Turner TN, Coe BP, Eichler EE 2017 Recurrent de novo mutations in neurodevelopmental disorders: properties and clinical implications. Genome Med. 9, 101 (10.1186/s13073-017-0498-x) 29179772 PMC5704398

[5] Lepeta K et al. 2016 Synaptopathies: synaptic dysfunction in neurological disorders—a review from students to students. J. Neurochem. 138, 785–805. (10.1111/jnc.13713) 27333343 PMC5095804

[6] Sudhof TC 2012 The presynaptic active zone. Neuron 75, 11–25. (10.1016/j.neuron.2012.06.012) 22794257 PMC3743085

[7] Rouach N, Avignone E, Meme W, Koulakoff A, Venance L, Blomstrand F, Giaume C 2002 Gap junctions and connexin expression in the normal and pathological central nervous system. Biol. Cell 94, 457–475. (10.1016/S0248-4900(02)00016-3) 12566220

[8] Bourne JN, Harris KM 2008 Balancing structure and function at hippocampal dendritic spines. Annu. Rev. Neurosci. 31, 47–67. (10.1146/annurev.neuro.31.060407.125646) 18284372 PMC2561948

[9] Pinto MJ, Almeida RD 2016 Puzzling out presynaptic differentiation. J. Neurochem. 139, 921–942. (10.1111/jnc.13702) 27315450

[10] Sheng M, Kim E 2011 The postsynaptic organization of synapses. Cold Spring Harb. Perspect. Biol. 3, a005678 (10.1101/cshperspect.a005678) 22046028 PMC3225953

[11] Missler M, Sudhof TC, Biederer T 2012 Synaptic cell adhesion. Cold Spring Harb. Perspect. Biol. 4, a005694 (10.1101/cshperspect.a005694) 22278667 PMC3312681

[12] Dieterich DC, Kreutz MR 2016 Proteomics of the synapse—a quantitative approach to neuronal plasticity. Mol. Cell. Proteomics 15, 368–381. (10.1074/mcp.R115.051482) 26307175 PMC4739661

[13] Wilhelm BG et al. 2014 Composition of isolated synaptic boutons reveals the amounts of vesicle trafficking proteins. Science 344, 1023–1028. (10.1126/science.1252884) 24876496

[14] Boyken J, Gronborg M, Riedel D, Urlaub H, Jahn R, Chua JJ 2013 Molecular profiling of synaptic vesicle docking sites reveals novel proteins but few differences between glutamatergic and GABAergic synapses. Neuron 78, 285–297. (10.1016/j.neuron.2013.02.027) 23622064

[15] Weingarten J et al. 2014 The proteome of the presynaptic active zone from mouse brain. Mol. Cell. Neurosci. 59, 106–118. (10.1016/j.mcn.2014.02.003) 24534009

[16] Ziv NE, Garner CC 2004 Cellular and molecular mechanisms of presynaptic assembly. Nat. Rev. Neurosci. 5, 385–399. (10.1038/nrn1370) 15100721

[17] Gray EG 1959 Axo-somatic and axo-dendritic synapses of the cerebral cortex: an electron microscope study. J. Anat. 93, 420–433. 13829103 PMC1244535

[18] Ichimura T, Hashimoto PH 1988 Structural components in the synaptic cleft captured by freeze-substitution and deep etching of directly frozen cerebellar cortex. J. Neurocytol. 17, 3–12. (10.1007/BF01735373) 3047323

[19] Zuber B, Nikonenko I, Klauser P, Muller D, Dubochet J 2005 The mammalian central nervous synaptic cleft contains a high density of periodically organized complexes. Proc. Natl Acad. Sci. USA 102, 19 192–19 197. (10.1073/pnas.0509527102) PMC1323199 16354833

[20] Jang S, Lee H, Kim E 2017 Synaptic adhesion molecules and excitatory synaptic transmission. Curr. Opin Neurobiol. 45, 45–50. (10.1016/j.conb.2017.03.005) 28390263

[21] Sudhof TC 2017 Synaptic neurexin complexes: a molecular code for the logic of neural circuits. Cell 171, 745–769. (10.1016/j.cell.2017.10.024) 29100073 PMC5694349

[22] Biederer T, Kaeser PS, Blanpied TA 2017 Transcellular nanoalignment of synaptic function. Neuron 96, 680–696. (10.1016/j.neuron.2017.10.006) 29096080 PMC5777221

[23] Frank RA, Grant SG 2017 Supramolecular organization of NMDA receptors and the postsynaptic density. Curr. Opin Neurobiol. 45, 139–147. (10.1016/j.conb.2017.05.019) 28577431 PMC5557338

[24] MacGillavry HD, Kerr JM, Blanpied TA 2011 Lateral organization of the postsynaptic density. Mol. Cell. Neurosci. 48, 321–331. (10.1016/j.mcn.2011.09.001) 21920440 PMC3216044

[25] Ko J, Choii G, Um JW 2015 The balancing act of GABAergic synapse organizers. Trends Mol. Med. 21, 256–268. (10.1016/j.molmed.2015.01.004) 25824541

[26] Craig AM, Kang Y 2007 Neurexin-neuroligin signaling in synapse development. Curr. Opin. Neurobiol. 17, 43–52. (10.1016/j.conb.2007.01.011) 17275284 PMC2820508

[27] Siddiqui TJ, Craig AM 2011 Synaptic organizing complexes. Curr. Opin. Neurobiol. 21, 132–143. (10.1016/j.conb.2010.08.016) 20832286 PMC3016466

[28] Tatti R, Haley MS, Swanson OK, Tselha T, Maffei A 2017 Neurophysiology and regulation of the balance between excitation and inhibition in neocortical circuits. Biol. Psychiatry 81, 821–831. (10.1016/j.biopsych.2016.09.017) 27865453 PMC5374043

[29] Ardiles AO, Grabrucker AM, Scholl FG, Rudenko G, Borsello T 2017 Molecular and cellular mechanisms of synaptopathies. Neural Plast. 2017, 2643943 (10.1155/2017/2643943) 28540088 PMC5429942

[30] Shrivastava AN, Aperia A, Melki R, Triller A 2017 Physico-pathologic mechanisms involved in neurodegeneration: misfolded protein-plasma membrane interactions. Neuron 95, 33–50. (10.1016/j.neuron.2017.05.026) 28683268

[31] Lashuel HA, Overk CR, Oueslati A, Masliah E 2013 The many faces of alpha-synuclein: from structure and toxicity to therapeutic target. Nat. Rev. Neurosci. 14, 38–48. (10.1038/nrn3406) 23254192 PMC4295774

[32] Goedert M 2001 The significance of tau and alpha-synuclein inclusions in neurodegenerative diseases. Curr. Opin. Genet. Dev. 11, 343–351. (10.1016/S0959-437X(00)00200-8) 11377973

[33] Tyebji S, Hannan AJ 2017 Synaptopathic mechanisms of neurodegeneration and dementia: Insights from Huntington's disease. Prog. Neurobiol. 153, 18–45. (10.1016/j.pneurobio.2017.03.008) 28377290

[34] Lim J, Yue Z 2015 Neuronal aggregates: formation, clearance, and spreading. Dev. Cell 32, 491–501. (10.1016/j.devcel.2015.02.002) 25710535 PMC4376477

[35] Wang YC, Lauwers E, Verstreken P 2017 Presynaptic protein homeostasis and neuronal function. Curr. Opin Genet. Dev. 44, 38–46. (10.1016/j.gde.2017.01.015) 28213157

[36] Olivero P et al. 2018 Proteostasis and mitochondrial role on psychiatric and neurodegenerative disorders: current perspectives. Neural Plast. 2018, 6798712 (10.1155/2018/6798712) 30050571 PMC6040257

[37] Klaips CL, Jayaraj GG, Hartl FU 2018 Pathways of cellular proteostasis in aging and disease. J. Cell Biol. 217, 51–63. (10.1083/jcb.201709072) 29127110 PMC5748993

[38] Kumar V, Sami N, Kashav T, Islam A, Ahmad F, Hassan MI 2016 Protein aggregation and neurodegenerative diseases: from theory to therapy. Eur. J. Med. Chem. 124, 1105–1120. (10.1016/j.ejmech.2016.07.054) 27486076

[39] Sweeney P et al. 2017 Protein misfolding in neurodegenerative diseases: implications and strategies. Transl. Neurodegener. 6, 6 (10.1186/s40035-017-0077-5) 28293421 PMC5348787

[40] Zoghbi HY, Bear MF 2012 Synaptic dysfunction in neurodevelopmental disorders associated with autism and intellectual disabilities. Cold Spring Harb. Perspect. Biol. 2012, a009886 (10.1101/cshperspect.a009886) PMC3282414 22258914

[41] Roeper J 2018 Closing gaps in brain disease-from overlapping genetic architecture to common motifs of synapse dysfunction. Curr. Opin. Neurobiol. 48, 45–51. (10.1016/j.conb.2017.09.007) 28968515

[42] van der Staay FJ, Arndt SS, Nordquist RE 2009 Evaluation of animal models of neurobehavioral disorders. Behav. Brain Funct. 5, 11 (10.1186/1744-9081-5-11) 19243583 PMC2669803

[43] Shi Y, Inoue H, Wu JC, Yamanaka S 2017 Induced pluripotent stem cell technology: a decade of progress. Nat. Rev. Drug Discov. 16, 115–130. (10.1038/nrd.2016.245) 27980341 PMC6416143

[44] Mertens J, Marchetto MC, Bardy C, Gage FH 2016 Evaluating cell reprogramming, differentiation and conversion technologies in neuroscience. Nat. Rev. Neurosci. 17, 424–437. (10.1038/nrn.2016.46) 27194476 PMC6276815

[45] Shi Y, Kirwan P, Smith J, MacLean G, Orkin SH, Livesey FJ 2012 A human stem cell model of early Alzheimer's disease pathology in Down syndrome. Sci. Transl. Med. 4(124r), a29. 10.1126/scitranslmed.3003771 PMC4129935 22344463

[46] Zhang Y et al. 2013 Rapid single-step induction of functional neurons from human pluripotent stem cells. Neuron 78, 785–798. (10.1016/j.neuron.2013.05.029) 23764284 PMC3751803

[47] Maroof AM et al. 2013 Directed differentiation and functional maturation of cortical interneurons from human embryonic stem cells. Cell stem cell 12, 559–572. (10.1016/j.stem.2013.04.008) 23642365 PMC3681523

[48] Yu DX et al. 2014 Modeling hippocampal neurogenesis using human pluripotent stem cells. Stem Cell Reports 2, 295–310. (10.1016/j.stemcr.2014.01.009) 24672753 PMC3964286

[49] Chambers SM, Fasano CA, Papapetrou EP, Tomishima M, Sadelain M, Studer L 2009 Highly efficient neural conversion of human ES and iPS cells by dual inhibition of SMAD signaling. Nat. Biotechnol. 27, 275–280. (10.1038/nbt.1529) 19252484 PMC2756723

[50] Lin L, Yuan J, Sander B, Golas MM 2015 In vitro differentiation of human neural progenitor cells into striatal GABAergic neurons. Stem Cells Transl. Med. 4, 775–788. (10.5966/sctm.2014-0083) 25972145 PMC4479615

[51] Weick JP 2016 Functional properties of human stem cell-derived neurons in health and disease. Stem Cells Int. 2016, 4190438. 27274733 10.1155/2016/4190438 PMC4870377

[52] Fink JJ, Levine ES 2018 Uncovering true cellular phenotypes: using induced pluripotent stem cell-derived neurons to study early insults in neurodevelopmental disorders. Front. Neurol. 9, 237. 29713304 10.3389/fneur.2018.00237 PMC5911479

[53] Gunhanlar N et al. 2018 A simplified protocol for differentiation of electrophysiologically mature neuronal networks from human induced pluripotent stem cells. Mol. Psychiatry 23, 1336–1344. (10.1038/mp.2017.56) 28416807 PMC5984104

[54] Frega M et al. 2017 Rapid neuronal differentiation of induced pluripotent stem cells for measuring network activity on micro-electrode arrays. J. Vis. Exp. **119**. 10.3791/54900 PMC5407693 28117798

[55] Bardy C et al. 2015 Neuronal medium that supports basic synaptic functions and activity of human neurons in vitro. Proc. Natl Acad. Sci. USA 112, E2725–E2734. (10.1073/pnas.1504393112) 25870293 PMC4443325

[56] Paull D et al. 2015 Automated, high-throughput derivation, characterization and differentiation of induced pluripotent stem cells. Nat. Methods 12, 885–892. (10.1038/nmeth.3507) 26237226 PMC13012702

[57] Juopperi TA, Kim WR, Chiang CH, Yu H, Margolis RL, Ross CA, Ming G, Song H 2012 Astrocytes generated from patient induced pluripotent stem cells recapitulate features of Huntington's disease patient cells. Mol. Brain 5, 17 (10.1186/1756-6606-5-17) 22613578 PMC3506453

[58] Abud EM et al. 2017 iPSC-derived human microglia-like cells to study neurological diseases. Neuron 94, 278–293. (10.1016/j.neuron.2017.03.042) 28426964 PMC5482419

[59] Kirwan P, Turner-Bridger B, Peter M, Momoh A, Arambepola D, Robinson HP, Livesey FJ 2015 Development and function of human cerebral cortex neural networks from pluripotent stem cells in vitro. Development 142, 3178–3187. (10.1242/dev.123851) 26395144 PMC4582178

[60] Chen TW et al. 2013 Ultrasensitive fluorescent proteins for imaging neuronal activity. Nature 499, 295–300. (10.1038/nature12354) 23868258 PMC3777791

[61] Avaliani N et al. 2014 Optogenetics reveal delayed afferent synaptogenesis on grafted human-induced pluripotent stem cell-derived neural progenitors. Stem Cells 32, 3088–3098. (10.1002/stem.1823) 25183299

[62] Virlogeux A et al. 2018 Reconstituting corticostriatal network on-a-chip reveals the contribution of the presynaptic compartment to Huntington's disease. Cell Rep. 22, 110–122. (10.1016/j.celrep.2017.12.013) 29298414

[63] Pruunsild P, Bengtson CP, Bading H 2017 Networks of cultured iPSC-derived neurons reveal the human synaptic activity-regulated adaptive gene program. Cell Rep. 18, 122–135. (10.1016/j.celrep.2016.12.018) 28052243 PMC5236011

[64] Fromer M et al. 2014 De novo mutations in schizophrenia implicate synaptic networks. Nature 506, 179–184. (10.1038/nature12929) 24463507 PMC4237002

[65] Studer L, Vera E, Cornacchia D 2015 Programming and reprogramming cellular age in the era of induced pluripotency. Cell Stem Cell 16, 591–600. (10.1016/j.stem.2015.05.004) 26046759 PMC4508309

[66] Brennand K et al. 2015 Phenotypic differences in hiPSC NPCs derived from patients with schizophrenia. Mol. Psychiatry 20, 361–368. (10.1038/mp.2014.22) 24686136 PMC4182344

[67] Miller JD et al. 2013 Human iPSC-based modeling of late-onset disease via progerin-induced aging. Cell Stem Cell 13, 691–705. (10.1016/j.stem.2013.11.006) 24315443 PMC4153390

[68] Nguyen HN et al. 2011 LRRK2 mutant iPSC-derived DA neurons demonstrate increased susceptibility to oxidative stress. Cell Stem Cell 8, 267–280. (10.1016/j.stem.2011.01.013) 21362567 PMC3578553

[69] Cooper O et al. 2012 Pharmacological rescue of mitochondrial deficits in iPSC-derived neural cells from patients with familial Parkinson's disease. Sci. Transl. Med. 4, 141ra90. 10.1126/scitranslmed.3003985 PMC3462009 22764206

[70] Kouroupi G et al. 2017 Defective synaptic connectivity and axonal neuropathology in a human iPSC-based model of familial Parkinson's disease. Proc. Natl Acad. Sci. USA 114, E3679–E3688. (10.1073/pnas.1617259114) 28416701 PMC5422768

[71] Lin L, Goke J, Cukuroglu E, Dranias MR, VanDongen AM, Stanton LW 2016 Molecular features underlying neurodegeneration identified through in vitro modeling of genetically diverse Parkinson's disease patients. Cell Rep. 15, 2411–2426. (10.1016/j.celrep.2016.05.022) 27264186

[72] Israel MA et al. 2012 Probing sporadic and familial Alzheimer's disease using induced pluripotent stem cells. Nature 482, 216–220. (10.1038/nature10821) 22278060 PMC3338985

[73] HD iPSC Consortium. 2017 Developmental alterations in Huntington's disease neural cells and pharmacological rescue in cells and mice. Nat. Neurosci. 20, 648–660. (10.1038/nn.4532) 28319609 PMC5610046

[74] Chen C et al. 2014 Role of astroglia in Down's syndrome revealed by patient-derived human-induced pluripotent stem cells. Nat. Commun. 5, 4430. 25034944 10.1038/ncomms5430 PMC4109022

[75] Windrem MS et al. 2017 Human iPSC glial mouse chimeras reveal glial contributions to schizophrenia. Cell Stem Cell 21, 195–208. (10.1016/j.stem.2017.06.012) 28736215 PMC5576346

[76] Marchetto MC, Muotri AR, Mu Y, Smith AM, Cezar GG, Gage FH 2008 Non-cell-autonomous effect of human SOD1 G37R astrocytes on motor neurons derived from human embryonic stem cells. Cell Stem Cell 3, 649–657. (10.1016/j.stem.2008.10.001) 19041781

[77] Pasca AM et al. 2015 Functional cortical neurons and astrocytes from human pluripotent stem cells in 3D culture. Nat. Methods 12, 671–678. (10.1038/nmeth.3415) 26005811 PMC4489980

[78] Jo J et al. 2016 Midbrain-like organoids from human pluripotent stem cells contain functional dopaminergic and neuromelanin-producing neurons. Cell Stem Cell 19, 248–257. (10.1016/j.stem.2016.07.005) 27476966 PMC5510242

[79] Pamies D et al. 2017 A human brain microphysiological system derived from induced pluripotent stem cells to study neurological diseases and toxicity. ALTEX 34, 362–376. (doi:10.14573/altex.1609122) 27883356 10.14573/altex.1609122 PMC6047513

[80] Lancaster MA et al. 2013 Cerebral organoids model human brain development and microcephaly. Nature 501, 373–379. (10.1038/nature12517) 23995685 PMC3817409

[81] Mariani J et al. 2015 FOXG1-dependent dysregulation of GABA/glutamate neuron differentiation in autism spectrum disorders. Cell 162, 375–390. (10.1016/j.cell.2015.06.034) 26186191 PMC4519016

[82] Qian X et al. 2016 Brain-region-specific organoids using mini-bioreactors for modeling ZIKV exposure. Cell 165, 1238–1254. (10.1016/j.cell.2016.04.032) 27118425 PMC4900885

[83] Yoon KJ et al. 2017 Zika-virus-encoded NS2A disrupts mammalian cortical neurogenesis by degrading adherens junction proteins. Cell Stem Cell 21, 349–358. (10.1016/j.stem.2017.07.014) 28826723 PMC5600197

[84] Birey F et al. 2017 Assembly of functionally integrated human forebrain spheroids. Nature 545, 54–59. (10.1038/nature22330) 28445465 PMC5805137

[85] Raja WK, Mungenast AE, Lin YT, Ko T, Abdurrob F, Seo J, Tsai L-H 2016 Self-organizing 3D human neural tissue derived from induced pluripotent stem cells recapitulate Alzheimer's disease phenotypes. PLoS ONE 11, e0161969 (10.1371/journal.pone.0161969) 27622770 PMC5021368

[86] Son MY, Sim H, Son YS, Jung KB, Lee MO, Oh JH, Chung S-K, Jung C-R, Kim J 2017 Distinctive genomic signature of neural and intestinal organoids from familial Parkinson's disease patient-derived induced pluripotent stem cells. Neuropathol. Appl. Neurobiol. 43, 584–603. (10.1111/nan.12396) 28235153

[87] Conforti P et al. 2018 Faulty neuronal determination and cell polarization are reverted by modulating HD early phenotypes. Proc. Natl Acad. Sci. USA 115, E762–E771. (10.1073/pnas.1715865115) 29311338 PMC5789931

[88] Di Lullo E, Kriegstein AR 2017 The use of brain organoids to investigate neural development and disease. Nat. Rev. Neurosci. 18, 573–584. (10.1038/nrn.2017.107) 28878372 PMC5667942

[89] Quadrato G, Brown J, Arlotta P 2016 The promises and challenges of human brain organoids as models of neuropsychiatric disease. Nat. Med. 22, 1220–1228. (10.1038/nm.4214) 27783065

[90] Bershteyn M, Nowakowski TJ, Pollen AA, Di Lullo E, Nene A, Wynshaw-Boris A, Kriegstein AR 2017 Human iPSC-derived cerebral organoids model cellular features of lissencephaly and reveal prolonged mitosis of outer radial glia. Cell Stem Cell 20, 435–449. (10.1016/j.stem.2016.12.007) 28111201 PMC5667944

[91] Ardhanareeswaran K, Mariani J, Coppola G, Abyzov A, Vaccarino FM 2017 Human induced pluripotent stem cells for modelling neurodevelopmental disorders. Nat. Rev. Neurol. 13, 265–278. (10.1038/nrneurol.2017.45) 28418023 PMC5782822

[92] Marchetto MC, Carromeu C, Acab A, Yu D, Yeo GW, Mu Y, Chen G, Gage FH, Muotri AR 2010 A model for neural development and treatment of Rett syndrome using human induced pluripotent stem cells. Cell 143, 527–539. (10.1016/j.cell.2010.10.016) 21074045 PMC3003590

[93] Shcheglovitov A et al. 2013 SHANK3 and IGF1 restore synaptic deficits in neurons from 22q13 deletion syndrome patients. Nature 503, 267–271. (10.1038/nature12618) 24132240 PMC5559273

[94] Sheridan SD, Theriault KM, Reis SA, Zhou F, Madison JM, Daheron L, Loring JF, Haggarty SJ 2011 Epigenetic characterization of the FMR1 gene and aberrant neurodevelopment in human induced pluripotent stem cell models of fragile X syndrome. PLoS ONE 6, e26203 (10.1371/journal.pone.0026203) 22022567 PMC3192166

[95] Doers ME et al. 2014 iPSC-derived forebrain neurons from FXS individuals show defects in initial neurite outgrowth. Stem Cells Dev. 23, 1777–1787. (10.1089/scd.2014.0030) 24654675 PMC4103262

[96] Lu P, Chen X, Feng Y, Zeng Q, Jiang C, Zhu X, Fan G, Xue Z 2016 Integrated transcriptome analysis of human iPS cells derived from a fragile X syndrome patient during neuronal differentiation. Sci. China Life Sci. 59, 1093–1105. (10.1007/s11427-016-0194-6) 27730449

[97] Boland MJ et al. 2017 Molecular analyses of neurogenic defects in a human pluripotent stem cell model of fragile X syndrome. Brain 140, 582–598. 28137726 10.1093/brain/aww357 PMC5837342

[98] Krey JF, Pasca SP, Shcheglovitov A, Yazawa M, Schwemberger R, Rasmusson R, Dolmetsch RE 2013 Timothy syndrome is associated with activity-dependent dendritic retraction in rodent and human neurons. Nat. Neurosci. 16, 201–209. (10.1038/nn.3307) 23313911 PMC3568452

[99] Tian Y, Voineagu I, Pasca SP, Won H, Chandran V, Horvath S, Dolmetsch RE, Geschwind DH 2014 Alteration in basal and depolarization induced transcriptional network in iPSC derived neurons from Timothy syndrome. Genome Med. 6, 75 (10.1186/s13073-014-0075-5) 25360157 PMC4213483

[100] Griesi-Oliveira K et al. 2015 Modeling non-syndromic autism and the impact of TRPC6 disruption in human neurons. Mol. Psychiatry 20, 1350–1365. (10.1038/mp.2014.141) 25385366 PMC4427554

[101] Brennand KJ et al. 2011 Modelling schizophrenia using human induced pluripotent stem cells. Nature 473, 221 (10.1038/nature09915) 21490598 PMC3392969

[102] Wen Z et al. 2014 Synaptic dysregulation in a human iPS cell model of mental disorders. Nature 515, 414–418. (10.1038/nature13716) 25132547 PMC4501856

[103] Yoon KJ et al. 2014 Modeling a genetic risk for schizophrenia in iPSCs and mice reveals neural stem cell deficits associated with adherens junctions and polarity. Cell Stem Cell 15, 79–91. (10.1016/j.stem.2014.05.003) 24996170 PMC4237009

[104] Lord C, Risi S, DiLavore PS, Shulman C, Thurm A, Pickles A 2006 Autism from 2 to 9 years of age. Arch. Gen. Psychiatry 63, 694–701. (10.1001/archpsyc.63.6.694) 16754843

[105] Sahin M, Sur M 2015 Genes, circuits, and precision therapies for autism and related neurodevelopmental disorders. Science 350, aab3897 (10.1126/science.aab3897) 26472761 PMC4739545

[106] Blanpied TA, Ehlers MD 2004 Microanatomy of dendritic spines: emerging principles of synaptic pathology in psychiatric and neurological disease. Biol. Psychiatry 55, 1121–1127. (10.1016/j.biopsych.2003.10.006) 15184030

[107] Irwin SA et al. 2001 Abnormal dendritic spine characteristics in the temporal and visual cortices of patients with fragile-X syndrome: a quantitative examination. Am. J. Med. Genet. 98, 161–167. (10.1002/1096-8628(20010115)98:2%3C161::AID-AJMG1025%3E3.0.CO;2-B) 11223852

[108] Kaufmann WE, Moser HW 2000 Dendritic anomalies in disorders associated with mental retardation. Cereb. Cortex 10, 981–991. (10.1093/cercor/10.10.981) 11007549

[109] Belichenko PV et al. 2004 Synaptic structural abnormalities in the Ts65Dn mouse model of Down syndrome. J. Comp. Neurol. 480, 281–298. (10.1002/cne.20337) 15515178

[110] Clement JP et al. 2012 Pathogenic SYNGAP1 mutations impair cognitive development by disrupting maturation of dendritic spine synapses. Cell 151, 709–723. (10.1016/j.cell.2012.08.045) 23141534 PMC3500766

[111] Humeau Y, Gambino F, Chelly J, Vitale N 2009 X-linked mental retardation: focus on synaptic function and plasticity. J. Neurochem. 109, 1–14. (10.1111/j.1471-4159.2009.05881.x) 19183273

[112] Huguet G, Ey E, Bourgeron T 2013 The genetic landscapes of autism spectrum disorders. Annu. Rev. Genomics Hum. Genet. 14, 191–213. (10.1146/annurev-genom-091212-153431) 23875794

[113] Srivastava AK, Schwartz CE 2014 Intellectual disability and autism spectrum disorders: causal genes and molecular mechanisms. Neurosci. Biobehav. Rev. 46, 161–174. (10.1016/j.neubiorev.2014.02.015) 24709068 PMC4185273

[114] De Rubeis S et al. 2014 Synaptic, transcriptional and chromatin genes disrupted in autism. Nature 515, 209–215. (10.1038/nature13772) 25363760 PMC4402723

[115] Heyes S, Pratt WS, Rees E, Dahimene S, Ferron L, Owen MJ, Dolphin AC 2015 Genetic disruption of voltage-gated calcium channels in psychiatric and neurological disorders. Prog. Neurobiol. 134, 36–54. (10.1016/j.pneurobio.2015.09.002) 26386135 PMC4658333

[116] Hagerman P 2013 Fragile X-associated tremor/ataxia syndrome (FXTAS): pathology and mechanisms. Acta Neuropathol. 126, 1–19. (10.1007/s00401-013-1138-1) 23793382 PMC3904666

[117] Etherton MR, Tabuchi K, Sharma M, Ko J, Sudhof TC 2011 An autism-associated point mutation in the neuroligin cytoplasmic tail selectively impairs AMPA receptor-mediated synaptic transmission in hippocampus. EMBO J. 30, 2908–2919. (10.1038/emboj.2011.182) 21642956 PMC3160244

[118] Pizzarelli R, Cherubini E 2013 Developmental regulation of GABAergic signalling in the hippocampus of neuroligin 3 R451C knock-in mice: an animal model of Autism. Front. Cell. Neurosci. 7, 85 (10.3389/fncel.2013.00085) 23761734 PMC3671185

[119] Tabuchi K, Blundell J, Etherton MR, Hammer RE, Liu X, Powell CM, Sudhof TC 2007 A neuroligin-3 mutation implicated in autism increases inhibitory synaptic transmission in mice. Science 318, 71–76. (10.1126/science.1146221) 17823315 PMC3235367

[120] Tandon R, Nasrallah HA, Keshavan MS 2009 Schizophrenia, ‘just the facts’ 4. Clinical features and conceptualization. Schizophr. Res. 110, 1–23. (10.1016/j.schres.2009.03.005) 19328655

[121] Kirov G et al. 2012 De novo CNV analysis implicates specific abnormalities of postsynaptic signalling complexes in the pathogenesis of schizophrenia. Mol. Psychiatry 17, 142–153. (10.1038/mp.2011.154) 22083728 PMC3603134

[122] Schizophrenia Working Group of the Psychiatric Genomics Consortium. 2014 Biological insights from 108 schizophrenia-associated genetic loci. Nature 511, 421–427. (10.1038/nature13595) 25056061 PMC4112379

[123] Carter CJ 2006 Schizophrenia susceptibility genes converge on interlinked pathways related to glutamatergic transmission and long-term potentiation, oxidative stress and oligodendrocyte viability. Schizophr. Res. 86, 1–14. (10.1016/j.schres.2006.05.023) 16842972

[124] Kirov G, Rujescu D, Ingason A, Collier DA, O'Donovan MC, Owen MJ 2009 Neurexin 1 (NRXN1) deletions in schizophrenia. Schizophr. Bull. 35, 851–854. (10.1093/schbul/sbp079) 19675094 PMC2728827

[125] Sekar A et al. 2016 Schizophrenia risk from complex variation of complement component 4. Nature 530, 177–183. (10.1038/nature16549) 26814963 PMC4752392

[126] Elmer BM, McAllister AK 2012 Major histocompatibility complex class I proteins in brain development and plasticity. Trends Neurosci. 35, 660–670. (10.1016/j.tins.2012.08.001) 22939644 PMC3493469

[127] Stefansson H et al. 2008 Large recurrent microdeletions associated with schizophrenia. Nature 455, 232–236. (10.1038/nature07229) 18668039 PMC2687075

[128] Bellucci A, Mercuri NB, Venneri A, Faustini G, Longhena F, Pizzi M, Missale C, Spano PF 2016 Review: Parkinson's disease: from synaptic loss to connectome dysfunction. Neuropathol. Appl. Neurobiol. 42, 77–94. (10.1111/nan.12297) 26613567

[129] Forner S, Baglietto-Vargas D, Martini AC, Trujillo-Estrada L, LaFerla FM 2017 Synaptic impairment in Alzheimer's disease: a dysregulated symphony. Trends Neurosci. 40, 347–357. (10.1016/j.tins.2017.04.002) 28494972

[130] Ross CA et al. 2014 Huntington disease: natural history, biomarkers and prospects for therapeutics. Nat. Rev. Neurol. 10, 204–216. (10.1038/nrneurol.2014.24) 24614516

[131] Curtis MA et al. 2003 Increased cell proliferation and neurogenesis in the adult human Huntington's disease brain. Proc. Natl Acad. Sci. USA 100, 9023–9027. (10.1073/pnas.1532244100) 12853570 PMC166431

[132] Ernst A et al. 2014 Neurogenesis in the striatum of the adult human brain. Cell 156, 1072–1083. (10.1016/j.cell.2014.01.044) 24561062

[133] Scahill RI et al. 2013 Clinical impairment in premanifest and early Huntington's disease is associated with regionally specific atrophy. Hum. Brain Mapp. 34, 519–529. 22102212 10.1002/hbm.21449 PMC6869979

[134] Niccolini F, Politis M 2014 Neuroimaging in Huntington's disease. World J. Radiol. 6, 301–312. (10.4329/wjr.v6.i6.301) 24976932 PMC4072816

[135] Paulsen JS et al. 2014 Clinical and biomarker changes in premanifest Huntington disease show trial feasibility: a decade of the PREDICT-HD study. Front. Aging Neurosci. 6, 78 (10.3389/fnagi.2014.00078) 24795630 PMC4000999

[136] Lee JK et al. 2012 Measures of growth in children at risk for Huntington disease. Neurology 79, 668–674. (10.1212/WNL.0b013e3182648b65) 22815549 PMC3414667

[137] Camnasio S et al. 2012 The first reported generation of several induced pluripotent stem cell lines from homozygous and heterozygous Huntington's disease patients demonstrates mutation related enhanced lysosomal activity. Neurobiol. Dis. 46, 41–51. (10.1016/j.nbd.2011.12.042) 22405424

[138] Liu GH et al. 2012 Progressive degeneration of human neural stem cells caused by pathogenic LRRK2. Nature 491, 603–607. (10.1038/nature11557) 23075850 PMC3504651

[139] Reinhardt P et al. 2013 Genetic correction of a LRRK2 mutation in human iPSCs links parkinsonian neurodegeneration to ERK-dependent changes in gene expression. Cell Stem Cell 12, 354–367. (10.1016/j.stem.2013.01.008) 23472874

[140] Schondorf DC et al. 2014 iPSC-derived neurons from GBA1-associated Parkinson's disease patients show autophagic defects and impaired calcium homeostasis. Nat. Commun. 5, 4028 (10.1038/ncomms5028) 24905578

[141] Woodard CM et al. 2014 iPSC-derived dopamine neurons reveal differences between monozygotic twins discordant for Parkinson's disease. Cell Rep. 9, 1173–1182. (10.1016/j.celrep.2014.10.023) 25456120 PMC4255586

[142] Shaltouki A, Sivapatham R, Pei Y, Gerencser AA, Momcilovic O, Rao MS, Zeng X 2015 Mitochondrial alterations by PARKIN in dopaminergic neurons using PARK2 patient-specific and PARK2 knockout isogenic iPSC lines. Stem Cell Reports 4, 847–859. (10.1016/j.stemcr.2015.02.019) 25843045 PMC4437475

[143] Ren Y, Jiang H, Hu Z, Fan K, Wang J, Janoschka S, Wang X, Ge S, Feng J 2015 Parkin mutations reduce the complexity of neuronal processes in iPSC-derived human neurons. Stem Cells 33, 68–78. (10.1002/stem.1854) 25332110 PMC4429885

[144] Burbulla LF et al. 2017 Dopamine oxidation mediates mitochondrial and lysosomal dysfunction in Parkinson's disease. Science 357, 1255–1261. (10.1126/science.aam9080) 28882997 PMC6021018

[145] Soldner F et al. 2011 Generation of isogenic pluripotent stem cells differing exclusively at two early onset Parkinson point mutations. Cell 146, 318–331. (10.1016/j.cell.2011.06.019) 21757228 PMC3155290

[146] Byers B et al. 2011 SNCA triplication Parkinson's patient's iPSC-derived DA neurons accumulate alpha-synuclein and are susceptible to oxidative stress. PLoS ONE 6, e26159. 22110584 10.1371/journal.pone.0026159 PMC3217921

[147] Ryan SD et al. 2013 Isogenic human iPSC Parkinson's model shows nitrosative stress-induced dysfunction in MEF2-PGC1alpha transcription. Cell 155, 1351–1364. (10.1016/j.cell.2013.11.009) 24290359 PMC4028128

[148] Chung CY et al. 2013 Identification and rescue of alpha-synuclein toxicity in Parkinson patient-derived neurons. Science 342, 983–987. (10.1126/science.1245296) 24158904 PMC4022187

[149] Flierl A et al. 2014 Higher vulnerability and stress sensitivity of neuronal precursor cells carrying an alpha-synuclein gene triplication. PLoS ONE 9, e112413. 25390032 10.1371/journal.pone.0112413 PMC4229205

[150] Oliveira LM et al. 2015 Elevated alpha-synuclein caused by SNCA gene triplication impairs neuronal differentiation and maturation in Parkinson's patient-derived induced pluripotent stem cells. Cell Death Dis. 6, e1994 (10.1038/cddis.2015.318) 26610207 PMC4670926

[151] Ryan T et al. 2018 Cardiolipin exposure on the outer mitochondrial membrane modulates alpha-synuclein. Nat. Commun. 9, 817. 29483518 10.1038/s41467-018-03241-9 PMC5827019

[152] Molero AE, Arteaga-Bracho EE, Chen CH, Gulinello M, Winchester ML, Pichamoorthy N, Gokhan S, Khodakhah K, Mehler MF 2016 Selective expression of mutant huntingtin during development recapitulates characteristic features of Huntington's disease. Proc. Natl Acad. Sci. USA 113, 5736–5741. (10.1073/pnas.1603871113) 27140644 PMC4878495

[153] Haremaki T, Deglincerti A, Brivanlou AH 2015 Huntingtin is required for ciliogenesis and neurogenesis during early Xenopus development. Dev. Biol. 408, 305–315. (10.1016/j.ydbio.2015.07.013) 26192473

[154] Ismailoglu I, Chen Q, Popowski M, Yang L, Gross SS, Brivanlou AH 2014 Huntingtin protein is essential for mitochondrial metabolism, bioenergetics and structure in murine embryonic stem cells. Dev. Biol. 391, 230–240. (10.1016/j.ydbio.2014.04.005) 24780625 PMC4109978

[155] Nguyen GD, Molero AE, Gokhan S, Mehler MF 2013 Functions of huntingtin in germ layer specification and organogenesis. PLoS ONE 8, e72698 (10.1371/journal.pone.0072698) 23967334 PMC3742581

[156] Sardo VL et al. 2012 An evolutionary recent neuroepithelial cell adhesion function of huntingtin implicates ADAM10-Ncadherin. Nat. Neurosci. 15, 713–721. (10.1038/nn.3080) 22466506

[157] McKinstry SU et al. 2014 Huntingtin is required for normal excitatory synapse development in cortical and striatal circuits. J. Neurosci. 34, 9455–9472. (10.1523/JNEUROSCI.4699-13.2014) 25009276 PMC4087216

[158] Molero AE, Gokhan S, Gonzalez S, Feig JL, Alexandre LC, Mehler MF 2009 Impairment of developmental stem cell-mediated striatal neurogenesis and pluripotency genes in a knock-in model of Huntington's disease. Proc. Natl Acad. Sci. USA 106, 21 900–21 905. (10.1073/pnas.0912171106) PMC2799796 19955426

[159] Braak H, Braak E, Yilmazer D, Schultz C, de Vos RA, Jansen EN 1995 Nigral and extranigral pathology in Parkinson's disease. J. Neural Transm. Suppl. 46, 15–31. 8821039

[160] Ulfig N, Braak E, Braak H 1989 Changes within the basal nucleus in Parkinson's disease. Prog. Clin. Biol. Res. 317, 493–500. 2690110

[161] Lees AJ, Hardy J, Revesz T 2009 Parkinson's disease. Lancet 373, 2055–2066. (10.1016/S0140-6736(09)60492-X) 19524782

[162] Schapira AH, Tolosa E 2010 Molecular and clinical prodrome of Parkinson disease: implications for treatment. Nat. Rev. Neurol. 6, 309–317. (10.1038/nrneurol.2010.52) 20479780

[163] Schirinzi T, Madeo G, Martella G, Maltese M, Picconi B, Calabresi P, Pisani A 2016 Early synaptic dysfunction in Parkinson's disease: insights from animal models. Mov. Disord. 31, 802–813. (10.1002/mds.26620) 27193205

[164] Calo L, Wegrzynowicz M, Santivanez-Perez J, Grazia Spillantini M 2016 Synaptic failure and alpha-synuclein. Mov. Disord. 31, 169–177. (10.1002/mds.26479) 26790375

[165] Kramer ML, Schulz-Schaeffer WJ 2007 Presynaptic alpha-synuclein aggregates, not Lewy bodies, cause neurodegeneration in dementia with Lewy bodies. J. Neurosci. 27, 1405–1410. (10.1523/JNEUROSCI.4564-06.2007) 17287515 PMC6673583

[166] Breza M et al. 2017 The different faces of the p.A53T alpha-synuclein mutation: a screening of Greek patients with parkinsonism and/or dementia. Neurosci. Lett. 672, 136–139. (10.1016/j.neulet.2017.12.015) 29233723

[167] Nemani VM, Lu W, Berge V, Nakamura K, Onoa B, Lee MK, Chaudhry FA, Nicoll RA, Edwards RH 2010 Increased expression of alpha-synuclein reduces neurotransmitter release by inhibiting synaptic vesicle reclustering after endocytosis. Neuron 65, 66–79. (10.1016/j.neuron.2009.12.023) 20152114 PMC3119527

[168] Koch JC et al. 2015 Alpha-synuclein affects neurite morphology, autophagy, vesicle transport and axonal degeneration in CNS neurons. Cell Death Dis. 6, e1811 (10.1038/cddis.2015.169) 26158517 PMC4650722

[169] Vargas KJ et al. 2017 Synucleins have multiple effects on presynaptic architecture. Cell Rep. 18, 161–173. (10.1016/j.celrep.2016.12.023) 28052246 PMC5510332

[170] Singleton AB, Farrer MJ, Bonifati V 2013 The genetics of Parkinson's disease: progress and therapeutic implications. Mov. Disord. 28, 14–23. (10.1002/mds.25249) 23389780 PMC3578399

[171] Petrucci S, Ginevrino M, Valente EM 2016 Phenotypic spectrum of alpha-synuclein mutations: new insights from patients and cellular models. Parkinsonism Relat. Disord. 22(Suppl. 1), S16–S20. (10.1016/j.parkreldis.2015.08.015) 26341711

[172] Torrent R, De Angelis Rigotti F, Dell'Era P, Memo M, Raya A, Consiglio A 2015 Using iPS cells toward the understanding of Parkinson's disease. J. Clin. Med. 4, 548–566. (10.3390/jcm4040548) 26239346 PMC4470155

[173] Dolt K S, Hammachi F, Kunath T 2017 Modeling Parkinson's disease with induced pluripotent stem cells harboring alpha-synuclein mutations. Brain Pathol. 27, 545–551. (10.1111/bpa.12526) 28585381 PMC8029042

[174] Zimprich A et al. 2004 Mutations in LRRK2 cause autosomal-dominant parkinsonism with pleomorphic pathology. Neuron 44, 601–607. (10.1016/j.neuron.2004.11.005) 15541309

[175] Taymans JM, Van den Haute C, Baekelandt V 2006 Distribution of PINK1 and LRRK2 in rat and mouse brain. J. Neurochem. 98, 951–961. (10.1111/j.1471-4159.2006.03919.x) 16771836

[176] Shin N et al. 2008 LRRK2 regulates synaptic vesicle endocytosis. Exp. Cell Res. 314, 2055–2065. (10.1016/j.yexcr.2008.02.015) 18445495

[177] Winner B et al. 2011 Adult neurogenesis and neurite outgrowth are impaired in LRRK2 G2019S mice. Neurobiol. Dis. 41, 706–716. (10.1016/j.nbd.2010.12.008) 21168496 PMC3059106

[178] Matta S et al. 2012 LRRK2 controls an EndoA phosphorylation cycle in synaptic endocytosis. Neuron 75, 1008–1021. (10.1016/j.neuron.2012.08.022) 22998870

[179] Parisiadou L et al. 2014 LRRK2 regulates synaptogenesis and dopamine receptor activation through modulation of PKA activity. Nat. Neurosci. 17, 367–376. (10.1038/nn.3636) 24464040 PMC3989289

[180] Sweet ES, Saunier-Rebori B, Yue Z, Blitzer RD 2015 The Parkinson's disease-associated mutation LRRK2-G2019S impairs synaptic plasticity in mouse hippocampus. J. Neurosci. 35, 11 190–11 195. (10.1523/JNEUROSCI.0040-15.2015) PMC4532754 26269629

[181] Koprich JB, Kalia LV, Brotchie JM 2017 Animal models of alpha-synucleinopathy for Parkinson disease drug development. Nat. Rev. Neurosci. 18, 515–529. (10.1038/nrn.2017.75) 28747776

[182] Polymeropoulos MH et al. 1997 Mutation in the alpha-synuclein gene identified in families with Parkinson's disease. Science 276, 2045–2047. (10.1126/science.276.5321.2045) 9197268

[183] Spillantini MG, Schmidt ML, Lee VM, Trojanowski JQ, Jakes R, Goedert M 1997 Alpha-synuclein in Lewy bodies. Nature 388, 839–840. (10.1038/42166) 9278044

[184] Appel-Cresswell S et al. 2013 Alpha-synuclein p.H50Q, a novel pathogenic mutation for Parkinson's disease. Mov. Disord. 28, 811–813. (10.1002/mds.25421) 23457019

[185] Kiely AP et al. 2013 Alpha- Synucleinopathy associated with G51D SNCA mutation: a link between Parkinson's disease and multiple system atrophy? Acta Neuropathol. 125, 753–769. (10.1007/s00401-013-1096-7) 23404372 PMC3681325

[186] Kruger R et al. 1998 Ala30Pro mutation in the gene encoding alpha-synuclein in Parkinson's disease. Nat. Genet. 18, 106–108. (10.1038/ng0298-106) 9462735

[187] Pasanen P et al. 2014 Novel alpha-synuclein mutation A53E associated with atypical multiple system atrophy and Parkinson's disease-type pathology. Neurobiol. Aging 35, 2180 (10.1016/j.neurobiolaging.2014.03.024) 24746362

[188] Proukakis C et al. 2013 A novel alpha-synuclein missense mutation in Parkinson disease. Neurology 80, 1062–1064. (10.1212/WNL.0b013e31828727ba) 23427326 PMC3653201

[189] Pasanen P et al. 2017 SNCA mutation p.Ala53Glu is derived from a common founder in the Finnish population. Neurobiol. Aging 50, 168 (10.1016/j.neurobiolaging.2016.10.014) 27838048

[190] Ibanez P, Lohmann E, Pollak P, Durif F, Tranchant C, Agid Y, Durr A, Brice A 2004 Absence of NR4A2 exon 1 mutations in 108 families with autosomal dominant Parkinson disease. Neurology 62, 2133–2134. (10.1212/01.WNL.0000127496.23198.75) 15184637

[191] Spira PJ, Sharpe DM, Halliday G, Cavanagh J, Nicholson GA 2001 Clinical and pathological features of a Parkinsonian syndrome in a family with an Ala53Thr alpha-synuclein mutation. Ann. Neurol. 49, 313–319. (10.1002/ana.67) 11261505

[192] Kotzbauer PT et al. 2004 Fibrillization of alpha-synuclein and tau in familial Parkinson's disease caused by the A53T alpha-synuclein mutation. Exp. Neurol. 187, 279–288. (10.1016/j.expneurol.2004.01.007) 15144854

[193] Fogel BL et al. 2012 RBFOX1 regulates both splicing and transcriptional networks in human neuronal development. Hum. Mol. Genet. 21, 4171–4186. (10.1093/hmg/dds240) 22730494 PMC3441119

[194] Soldner F et al. 2009 Parkinson's disease patient-derived induced pluripotent stem cells free of viral reprogramming factors. Cell 136, 964–977. (10.1016/j.cell.2009.02.013) 19269371 PMC2787236

[195] Duda JE, Giasson BI, Mabon ME, Miller DC, Golbe LI, Lee VM, Trojanowski JQ 2002 Concurrence of alpha-synuclein and tau brain pathology in the Contursi kindred. Acta Neuropathol. 104, 7–11. (10.1007/s00401-002-0563-3) 12070658

[196] Kriks S et al. 2011 Dopamine neurons derived from human ES cells efficiently engraft in animal models of Parkinson's disease. Nature 480, 547–551. (10.1038/nature10648) 22056989 PMC3245796

[197] Scott DA, Tabarean I, Tang Y, Cartier A, Masliah E, Roy S 2010 A pathologic cascade leading to synaptic dysfunction in alpha-synuclein-induced neurodegeneration. J. Neurosci. 30, 8083–8095. (10.1523/JNEUROSCI.1091-10.2010) 20554859 PMC2901533

[198] Victor MB et al. 2018 Striatal neurons directly converted from Huntington's disease patient fibroblasts recapitulate age-associated disease phenotypes. Nat. Neurosci. 21, 341–352. (10.1038/s41593-018-0075-7) 29403030 PMC5857213

[199] Hollander E, Wang AT, Braun A, Marsh L 2009 Neurological considerations: autism and Parkinson's disease. Psychiatry Res. 170, 43–51. (10.1016/j.psychres.2008.07.014) 19815296

[200] Teitelbaum P, Teitelbaum O, Nye J, Fryman J, Maurer RG 1998 Movement analysis in infancy may be useful for early diagnosis of autism. Proc. Natl Acad. Sci. USA 95, 13 982–13 987. (10.1073/pnas.95.23.13982) 9811912 PMC25000

[201] Starkstein S, Gellar S, Parlier M, Payne L, Piven J 2015 High rates of parkinsonism in adults with autism. J. Neurodev. Disord. 7, 29 (10.1186/s11689-015-9125-6) 26322138 PMC4553212

[202] Scheuerle A, Wilson K 2011 PARK2 copy number aberrations in two children presenting with autism spectrum disorder: further support of an association and possible evidence for a new microdeletion/microduplication syndrome. Am. J. Med. Genet. B Neuropsychiatr. Genet. 156B, 413–420. (10.1002/ajmg.b.31176) 21360662

[203] Sauerzopf U, Sacco R, Novarino G, Niello M, Weidenauer A, Praschak-Rieder N, Sitte H, Willeit M 2017 Are reprogrammed cells a useful tool for studying dopamine dysfunction in psychotic disorders? A review of the current evidence. Eur. J. Neurosci. 45, 45–57. (10.1111/ejn.13418) 27690184 PMC5811827

[204] Head E, Powell D, Gold BT, Schmitt FA 2012 Alzheimer's disease in Down syndrome. Eur. J. Neurodegener. Dis. 1, 353–364. 25285303 PMC4184282

[205] Briggs JA et al. 2013 Integration-free induced pluripotent stem cells model genetic and neural developmental features of down syndrome etiology. Stem Cells 31, 467–478. (10.1002/stem.1297) 23225669

[206] Weick JP et al. 2013 Deficits in human trisomy 21 iPSCs and neurons. Proc. Natl Acad. Sci. USA 110, 9962–9967. (10.1073/pnas.1216575110) 23716668 PMC3683748

[207] Redies C, Hertel N, Hubner CA 2012 Cadherins and neuropsychiatric disorders. Brain Res. 1470, 130–144. (10.1016/j.brainres.2012.06.020) 22765916

[208] Um JW et al. 2014 Structural basis for LAR-RPTP/Slitrk complex-mediated synaptic adhesion. Nat. Commun. 5, 5423 (10.1038/ncomms6423) 25394468

[209] Zaltieri M et al. 2015 alpha-synuclein and synapsin III cooperatively regulate synaptic function in dopamine neurons. J. Cell Sci. 128, 2231–2243. (10.1242/jcs.157867) 25967550

[210] Chen Q et al. 2009 Association and expression study of synapsin III and schizophrenia. Neurosci. Lett. 465, 248–251. (10.1016/j.neulet.2009.09.032) 19766700 PMC2777515

[211] Greenwood TA et al. 2016 Genetic assessment of additional endophenotypes from the Consortium on the Genetics of Schizophrenia Family Study. Schizophr. Res. 170, 30–40. (10.1016/j.schres.2015.11.008) 26597662 PMC4707095

[212] Chaudhry M, Wang X, Bamne MN, Hasnain S, Demirci FY, Lopez OL, Kamboh MI 2015 Genetic variation in imprinted genes is associated with risk of late-onset Alzheimer's disease. J. Alzheimers Dis. 44, 989–994. (10.3233/JAD-142106) 25391383 PMC4324355

[213] Tarabeux J et al. 2011 Rare mutations in N-methyl-D-aspartate glutamate receptors in autism spectrum disorders and schizophrenia. Transl. Psychiatry 1, e55 (10.1038/tp.2011.52) 22833210 PMC3309470

